# Optimal nonlinear information processing capacity in delay-based reservoir computers

**DOI:** 10.1038/srep12858

**Published:** 2015-09-11

**Authors:** Lyudmila Grigoryeva, Julie Henriques, Laurent Larger, Juan-Pablo Ortega

**Affiliations:** 1 Laboratoire de Mathématiques de Besançon, UMR CNRS 6623, Université de Franche-Comté, UFR des Sciences et Techniques. 16, route de Gray. F-25030 Besançon cedex. France; 2 Cegos Deployment. 11, rue Denis Papin. F-25000 Besançon; 3 FEMTO-ST, UMR CNRS 6174, Optics Department, Université de Franche-Comté, UFR des Sciences et Techniques. 15, Avenue des Montboucons. F-25000 Besançon cedex. France; 4 Centre National de la Recherche Scientifique, Laboratoire de Mathématiques de Besançon, UMR CNRS 6623, Université de Franche-Comté, UFR des Sciences et Techniques. 16, route de Gray. F-25030 Besançon cedex. France

## Abstract

Reservoir computing is a recently introduced brain-inspired machine learning paradigm capable of excellent performances in the processing of empirical data. We focus in a particular kind of time-delay based reservoir computers that have been physically implemented using optical and electronic systems and have shown unprecedented data processing rates. Reservoir computing is well-known for the ease of the associated training scheme but also for the problematic sensitivity of its performance to architecture parameters. This article addresses the reservoir design problem, which remains the biggest challenge in the applicability of this information processing scheme. More specifically, we use the information available regarding the optimal reservoir working regimes to construct a functional link between the reservoir parameters and its performance. This function is used to explore various properties of the device and to choose the optimal reservoir architecture, thus replacing the tedious and time consuming parameter scannings used so far in the literature.

The increase in need for information processing capacity, as well as the physical limitations of the Turing or von Neumann machine methods implemented in most computational systems, have motivated the search for new brain-inspired solutions some of which present an outstanding potential. An important direction in this undertaking is based on the use of the intrinsic information processing abilities of dynamical systems^1^ which opens the door to high performance physical realizations whose behavior is ruled by these structures^2^,^3^.

The contributions in this paper take place in a specific implementation of this idea that is obtained as a melange of a recently introduced machine learning paradigm known under the name of **reservoir computing (RC)**^4^,^5^,^6^,^7^,^8^,^9^,^10^ with a realization based on the sampling of the solution of a time-delay differential equation^11^,^12^. We refer to this combination as **time-delay reservoirs (TDRs)**. Physical implementations of this scheme carried out with dedicated hardware are already available and have shown excellent performances in the processing of empirical data: spoken digit recognition^13^,^14^,^15^,^16^,^17^, the NARMA model identification task^11^,^18^, continuation of chaotic time series, and volatility forecasting^19^. A recent example that shows the potential of this combination is the results in^17^ where an optoelectronic implementation of a TDR is capable of achieving the lowest documented error in the speech recognition task at unprecedented speed in an experiment design in which digit and speaker recognition are carried out in parallel.

A major advantage of RC is the linearity of its training scheme. This choice makes its implementation easy when compared to more traditional machine learning approaches like recursive neural networks, which usually require the solution of convoluted and sometimes ill-defined optimization problems. In exchange, as it can be seen in most of the references quoted above, the system performance is not robust with respect to the choice of the parameter values ***θ*** of the nonlinear kernel used to construct the RC (see below). More specifically, small deviations from the optimal parameter values can seriously degrade the performance and moreover, the optimal parameters are highly dependent on the task at hand. This observation makes the kernel parameter optimization a very important step in the RC design and has motivated the introduction of alternative parallel-based architectures^19^,^20^ to tackle this difficulty.

The main contribution of this paper is the introduction of an approximated model that, to our knowledge, provides the first rigorous analytical description of the delay-based RC performance. This powerful theoretical tool can be used to systematically study the delay-based RC properties and to replace the trial and error approach in the choice of architecture parameters by well structured optimization problems. This method simplifies enormously the implementation effort and sheds new light on the mechanisms that govern this information processing technique.

TDRs are based on the interaction of the time-dependent input signal 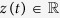 that we are interested in with the solution space of a time-delay differential equation of the form


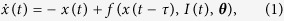


where *f* is a nonlinear smooth function (we call it **nonlinear kernel**) that depends on the *K* parameters in the vector 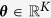, *τ* > 0 is the **delay**, 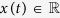, and 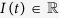 is obtained using a temporal multiplexing over the delay period of the input signal *z*(*t*) that we explain later on. We note that, even though the differential equation takes values in the real line, its solution space is infinite dimensional since an entire function 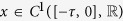 needs to be specified in order to initialize it. The choice of nonlinear kernel is determined by the intended physical implementation of the computing system; we focus on two parametric sets of kernels that have already been explored in the literature, namely, the Mackey-Glass^21^ and the Ikeda^22^ families. These kernels were used for reservoir computing purposes in the RC electronic and optic realizations in^14^ and^15^, respectively.

In order to visualize the TDR construction using a neural networks approach it is convenient, as in^12^,^14^, to consider the Euler time-discretization of (1) with integration step 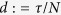, namely,


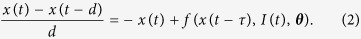


The design starts with the choice of a number 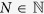 of **virtual neurons** and of an adapted **input mask** 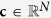. Next, the input signal *z*(*t*) at a given time *t* is multiplexed over the delay period by setting 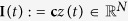 (see Module A in Fig. 1). We then organize it, as well as the solutions of (2), in **neuron layers x**(*t*) parametrized by a discretized time 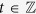 by setting


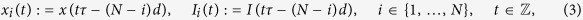


where *x*_*i*_(*t*) and *I*_*i*_(*t*) stand for the *i*th-components of the vectors **x**(*t*) and **I**(*t*), respectively, with 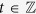. We say that *x*_*i*_(*t*) is the ***i*th neuron value of the** ***t*****th layer of the reservoir** and *d* is referred to as the **separation between neurons**. With this convention, the solutions of (2) are described by the following recursive relation:


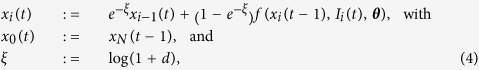


that shows how, as depicted in Module B in Fig. 1, any neuron value is a convex linear combination of the previous neuron value in the same layer and a nonlinear function of both the same neuron value in the previous layer and the input. The weights of this combination are determined by the separation between neurons; when the distance *d* is small, the neuron value *x*_*i*_(*t*) is mainly influenced by the previous neuron value *x*_*i*−1_(*t*), while large distances between neurons give predominance to the previous layer and foster the input gain. The recursions (4) uniquely determine a smooth map 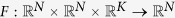 that specifies the neuron values as a recursion on the neuron layers via an expression of the form


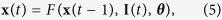


where *F* is constructed out of the nonlinear kernel map *f* that depends on the *K* parameters in the vector ***θ***; *F* is referred to as the **reservoir map**.

The construction of the TDR computer is finalized by connecting, as in Module C of Fig. 1, the reservoir output to a linear readout 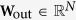 that is calibrated using a training sample by minimizing the associated task mean square error via a linear regression. We will refer to the Module B in Fig. 1 as the **reservoir** or the **time-delay reservoir** (TDR) and to the collection of the three modules as the **reservoir computer** (RC) or the **TDR computer**. A TDR based on the direct sampling of the solutions of (1) will be called a **continuous time TDR** and those based on the recursion (5) will be referred to as **discrete time TDRs**.

As we already mentioned, the performance of the RC for a given task is much dependent on the value of the kernel parameters ***θ*** and, in some cases, on the entries of the input mask **c** used for signal multiplexing. When comparing RC to standard neural networks and thinking of it as a machine learning paradigm, the RC training phase can be assimilated to the determination of both the linear readout **W**_out_ (straightforward in this case using a linear regression) and the optimal parameters ***θ***. Unlike the situation encountered in the neural networks context for which efficient training algorithms have been developed over the years (see^23^ for a particularly good performing example), the optimal parameters ***θ*** are usually determined in the RC context by trial and error or using computationally costly systematic scannings that are by far the biggest burden at the time of adapting the RC to a new task.

In this paper we construct an approximate model that we use to establish a functional link between the RC performance and the parameters ***θ*** and the input mask values **c**. Given a specific task, this explicit expression can be used to find appropriate parameter and mask values by solving a well structured and algorithmically convenient optimization problem that readily provides them.

The construction of this approximated formula is based on the observation that the optimal RC performance is always obtained when the TDR is working in a **stable unimodal regime**, that is, the reservoir is initialized at a stable equilibrium of the autonomous system (*I*(*t*) = 0) associated to (1) and the mean and variance of the input signal *I*(*t*) are designed using the input mask **c** so that the reservoir output remains around it and does not visit other stable equilibria or dynamical elements. In the next section we provide empirical and theoretical arguments for this claim. The performance measures that we consider in our study are the nonlinear memory capacities introduced in^24^ as a generalization of the linear concept proposed in^25^,^26^,^27^,^28^.

## Results

### Optimal performance: stability and unimodality

#### Stability and the reservoir defining properties

The estimations of the RC performance using the nonlinear memory capacity that we present later on, consist of approximating the reservoir by its partial linearization at the level of the delayed self feedback term and of respecting the nonlinearity in the input injection. This approach is only acceptable when the optimal dynamical regime that we are interested in, remains close to a given point. A natural candidate for such qualitative behavior could be obtained by initializing the reservoir at an asymptotically stable equilibrium of the autonomous system associated to (1) and by controlling the mean and the variance of the input signal *I*(*t*) so that the reservoir output remains close to it. This equilibrium point can be interpreted as an input bias that, for general RCs, is one of the most commonly optimized parameters.

There is both theoretical and empirical evidence that suggests that optimal performance is obtained for the RCs that we are interested in when working in a statistically stationary regime around a stable equilibrium. Indeed, one of the defining features of RC, namely the **echo state property**, is materialized for general RCs by enforcing that the spectral radius of the internal connectivity matrix of the reservoir is smaller than one^5^,^6^,^10^, which is the critical stability value for a quiescent state of the network when operating autonomously (without external injected information). It is well-known that the translation of this condition for TDRs implies parameter settings that ensure the existence of a stable state of (1) when *I*(*t*) is set to zero. This feature typically relates to gains of the feedback smaller than the Hopf threshold of the delay dynamics or, equivalently, to a sufficiently low feedback rate so that self-sustained oscillations are avoided.

Asymptotic stability is closely related with the so-called **fading memory property**^6^,^29^: the impact of any past injected input necessarily vanishes after a transient whose duration is typically of the order of the absolute value of the inverse of the smallest negative real part in the Lyapunov exponents. When the feedback gain is set too close to zero, the RC does not exhibit a long enough transient and thus presents an intrinsic memory that is too short to secure the self mixing of the temporal information necessary for its processing. On the other hand, if the feedback gain is set too close to the instability threshold, the input information flow requires too much time to vanish and hence the fading memory property is poorly satisfied. We recall the well known fact (see Section 8.2 in^29^) that the fading memory property can be realized by input-output systems generated by time-delay differential equations only when these exhibit a unique stable equilibrium.

In the context of recent successful physical realizations of RC, experimental parameters are systematically chosen so that the conditions described above are satisfied. Indeed, in^14^,^15^ these conditions are ensured via a proper tuning of the gain of the delayed feedback function. This approach differs from the one in^17^, where the conditions are met by choosing a laser injection current strictly smaller but close to the lasing threshold, as well as by using a moderate feedback, which prevents eventual self sustained external cavity mode oscillations. An additional important observation suggested by all these experimental setups is the need for a nonlinearity at the level of the input injection. In^14^,^15^ this feature is obtained using a strong enough input signal amplitude and via the transformation associated to the nonlinear delayed feedback. In^17^ the delayed feedback is linear but an external Mach-Zehnder modulator is used that implicitly provides a nonlinear transformation of the input signal as it is optically seeded through the nonlinear electro-optic modulation transfer function of the Mach-Zehnder.

We conclude by emphasizing that, even though optimal performance is attained when working in a statistically stationary regime around a stable equilibrium for the specific time-delay RCs that we are using, this is not a general feature that applies to all RCs or other network based computational paradigms. Indeed, recent works^30^ prove the existence of RCs (random Boolean networks in the case of^30^) that exhibit optimal performance when operating in a chaotic regime; more generally, this fact has also been observed in certain recurrent neural networks^31^.

#### Stability analysis of the time-delay reservoir

Due to the central role played by stability in our discussion, we now carefully analyze various sufficient conditions that ensure that the RC is functioning in a stable regime. All the statements that follow are carefully proved in the Supplementary Material section. Consider first an equilibrium 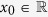 of the continuous time model (1) working in autonomous regime, that is, we set *I*(*t*) = 0. It can be shown using a Lyapunov-Krasovskiy-type analysis^32^,^33^ that the asymptotic stability of *x*_0_ is guaranteed whenever there exists an *ε* > 0 and a constant 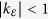 such that either

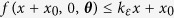
 for all 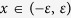, or
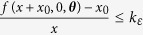
 for all 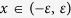.

The first condition can be used to prove the stability of equilibria exhibited by TDRs created using concave (but not necessarily differentiable) nonlinear kernels. As to the second one, it shows that if *f* is differentiable at *x*_0_ then this point is stable as long as 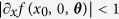, with 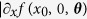 the first derivative of the nonlinear kernel *f* in (1) with respect to the first argument at the point (*x*_0_, 0, ***θ***).

The stability study can also be carried out by working with the discrete-time approximation (5) of the TDR which is determined by the reservoir map 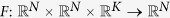. More specifically, it can be shown that 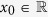 is an equilibrium of (1) if and only if 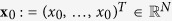 is a fixed point of (5). The asymptotic stability of this fixed point is ensured whenever the linearization *D*_**x**_*F*(**x**_0_, **0**_*N*_, ***θ***), which is a *N* × *N* matrix that will be referred to as the **connectivity matrix**, has a spectral radius smaller than one. Since it is not possible to compute the eigenvalues of *D*_**x**_*F*(**x**_0_, **0**_*N*_, ***θ***) for an arbitrary number of neurons *N*, we are hence obliged to proceed by finding estimations for the Cauchy bound^34^ of its characteristic polynomial or by bounding the spectral radius *ρ*(*D*_**x**_*F*(**x**_0_, **0**_*N*_, ***θ***)) using either a matrix norm or the Gershgorin discs^35^. An in-depth study of all these options showed that it is the use of the maximum row sum matrix norm 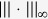 that yields the best stability bounds via the following statement:


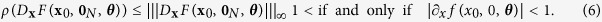


Notice that this remarkable result puts together the stability conditions for the continuous and discrete time systems.

As an example of application of these results, consider the Mackey-Glass nonlinear kernel^21^


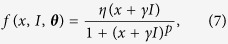


where the parameter 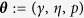 is a three tuple of real values; *γ* is usually referred to as the **input gain** and *η* the **feedback gain**. When this prescription is used in (1) in the autonomous regime, that is, *I*(*t*) = 0, the associated dynamical system exhibits two families of equilibria *x*_0_ parametrized by *η*, namely, *x*_0_ = 0 and the roots of 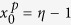. For example, in the case *p* = 2, two distinct cases arise: when *η* < 1 there is a unique equilibrium at the origin which is stable as long as 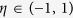. When *η* > 1 two other equilibria appear at *x*_0_ = ±(*η* − 1)^1/2^ which are stable whenever *η* < 3. These statements are proved in Corollary D.6 of the Supplementary Material. Analogous statements for the Ikeda kernel^22^ *f*(*x*, *I*, ***θ***) = *η* sin^2^(*x* + *γI* + *ϕ*), 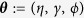 can be found in Corollary D.7 of the Supplementary Material. A particularly convenient sufficient condition is 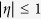 that simultaneously ensures stability and unimodality (existence of a single stable equilibrium).

#### Empirical evidence

**Figure 2:**
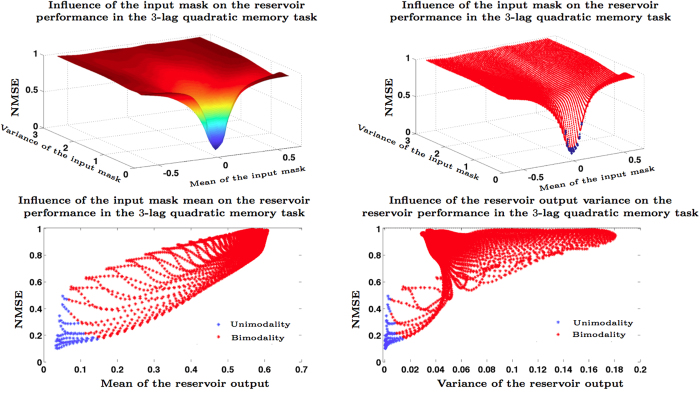
Behavior of the reservoir performance in a quadratic memory task as a function of the mean and the variance of the input mask. The modification of any of these two parameters influences how the reservoir dynamics separates from the stable equilibrium. The top panels show how the performance degrades very quickly as soon as the mean and the variance of the input mask (and hence of the input signal) separate from zero. The bottom panels depict the reservoir performance as a function of the various output means and variances obtained when changing the input means and variances. In the top right and bottom panels we have indicated with red markers the cases in which the reservoir visits the stability basin of a contiguous stable equilibrium hence showing how unimodality is associated to optimal performance.

In order to confirm these theoretical and experimental arguments, we have carried out several numerical simulations in which we studied the RC performance in terms of the dynamical regime of the reservoir at the time of carrying out various nonlinear memory tasks. More specifically, we construct a reservoir using the Ikeda nonlinear kernel with *N* = 20, *d* = 0.2581, *η* = 1.2443, *γ* = 1.4762, and *ϕ* = 0.1161. The equilibria of the associated autonomous system are given by the points *x*_0_ where the curves *y* = *x* and *y* = *η* sin^2^(*x* + *ϕ*) intersect. With this parameter values, intersections take place at *x*_0_ = 0.0244, *x*_0_ = 0.9075, and *x*_0_ = 1.063, which makes multi modality possible. As it can be shown with the results in the Supplementary Material section (see Corollary D.7), the first and the third equilibria are stable. In order to verify that the optimal performance is obtained when the RC operates in the neighborhood of a stable equilibrium, we study the normalized mean square error (NMSE) exhibited by a TDR initialized at *x*_0_ = 0.0244 when we present to it a quadratic memory task. More specifically, we inject in a TDR an independent and identically normally distributed signal *z*(*t*) with mean zero and variance 10^−4^ and we then train a linear readout **W**_out_ (obtained with a ridge penalization of λ = 10^−15^) in order to recover the quadratic function *z*(*t* − 1)^2^ + *z*(*t* − 2)^2^ + *z*(*t* − 3)^2^ out of the reservoir output. The top left panel in Fig. 2 shows how the NMSE behaves as a function of the mean and the variance of the input mask **c**. It is clear that by modifying any of these two parameters we control how far the reservoir dynamics separates from the stable equilibrium, which we quantitatively evaluate in the two bottom panels by representing the RC performance in terms of the mean and the variance of the resulting reservoir output. Both panels depict how the injection of a signal slightly shifted in mean or with a sufficiently high variance results in reservoir outputs that separate from the stable equilibrium and in a severely degraded performance. An important factor in this deterioration seems to be the multi modality, that is, if the shifting in mean or the input signal variance are large enough then the reservoir output visits the stability basin of the other stable point placed at *x*_0_ = 1.063; in the top right and bottom panels we have marked with red color the values for which bimodality has occurred so that the negative effect of this phenomenon is noticeable. In the Supplementary Material section we illustrate how the behavior that we just described is robust with respect to the choice of nonlinear kernel and is similar when the experiment is carried out using the Mackey-Glass function.

### The approximating model and the nonlinear memory capacity of the reservoir computer

The findings just presented have major consequences in the theoretical tools available for the evaluation of the RC performance. Indeed, since we now know that optimal operation is attained when the TDR functions in a unimodal fashion around an asymptotically stable steady state, we can approximate it by its partial linearization with respect to the delayed self feedback term at that point and keeping the nonlinearity for the input injection. For statistically independent input signals of the type used to compute nonlinear memory capacities of the type introduced in^24^, this approximation allows us to visualize the TDR as a *N*-dimensional (*N* is the number of neurons) vector autoregressive stochastic process of order one^36^ (we denote it as VAR(1)) for which the value of the associated nonlinear memory capacities can be explicitly computed. As we elaborate later on in the discussion, the quality of this approximation at the time of evaluating the memory capacities of the original system is excellent and the resulting function can be hence used for RC optimization purposes regarding the nonlinear kernel parameter values ***θ*** and the input mask **c**.

Consider a stable equilibrium 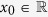 of the autonomous system associated to (1) or, equivalently, a stable fixed point of (5) of the form 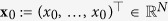. If we approximate (5) by its partial linearization at **x**_0_ with respect to the delayed self feedback and by the *R*-order Taylor series expansion of the functional that describes the signal injection, we obtain an expression of the form:


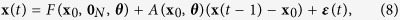


where 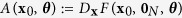 is the linear connectivity matrix and *ε*(*t*) is given by:


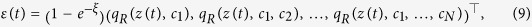


with


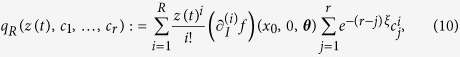


and 
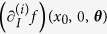
 is the *i*th order partial derivative of the nonlinear kernel *f* with respect to the second argument *I*(*t*), evaluated at the point (*x*_0_, 0, ***θ***).

If we now use as input signal *z*(*t*) independent and identically distributed random variables with mean 0 and variance 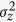 (we denote it by 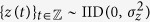) then the recursion (8) makes the reservoir layer dynamics 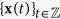 into a discrete time random process that, as we show in what follows, is the solution of a *N*-dimensional vector autoregressive model of order 1 (VAR(1)). Indeed, it is easy to see that the assumption 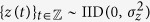 implies that 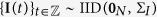, with 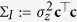, and that 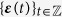 is a family of *N*-dimensional independent and identically distributed random variables with mean ***μ***_*ε*_ and covariance matrix 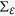 given by the following expressions:


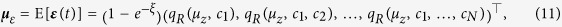


where the polynomial *q*_*R*_ is the same as in (10) and where we use the convention that the powers 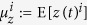, for any 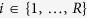 and with E[·] denoting the mathematical expectation. Additionally, 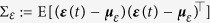 has entries determined by the relation:


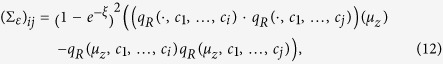


where the first summand stands for the multiplication of the polynomials 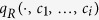 and 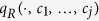 and the subsequent evaluation of the resulting polynomial at *μ*_*z*_, and the second one is made out of the multiplication of the evaluation of the two polynomials.

Using these observations, we can consider (8) as the prescription of a VAR(1) model driven by the independent noise 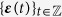. If the nonlinear kernel *f* satisfies the generic condition that the polynomial in *u* given by 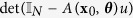, does not have roots in and on the complex unit circle, then (8) has a second order stationary solution^36^ 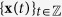 with time-independent mean given by


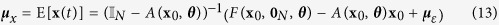


and an also time independent autocovariance function 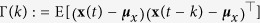, 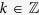, recursively determined the Yule-Walker equations (see^36^ for a detailed presentation). Indeed, Γ(0) is given by the vectorized equality:


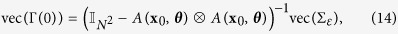


which determines the higher order autocovariances via the relation Γ(*k*) = *A*(**x**_0_, ***θ***)Γ(*k* − 1) and the identity Γ(−*k*) = Γ(*k*)^⊤^. As we explain in the following paragraphs, the moments (11), (13), and (14) are all that is needed in order to characterize the memory capacities of the RC.

A ***h*****-lag memory task** is determined by a (in general nonlinear) function 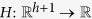 that is used to generate a one-dimensional signal 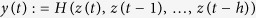 out of the reservoir input. Given a TDR computer, the optimal linear readout **W**_out_ adapted to the memory task *H* is given by the solution of a ridge linear regression problem with regularization parameter 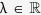 (usually tuned during the training phase via cross-validation) in which the covariates are the neuron values corresponding to the reservoir output and the explained variables are the values {*y*(*t*)} of the memory task function. The *H*-memory capacity *C*_*H*_(***θ***, **c**, λ) of the TDR computer under consideration characterized by a nonlinear kernel *f* with parameters ***θ***, an input mask **c**, and a regularizing ridge parameter λ is defined as one minus the normalized mean square error committed at the time of accomplishing the memory task *H*. When the reservoir is approximated by a VAR(1) process, then the corresponding ***H*****-memory capacity** is given by


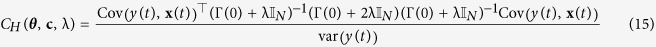


The developments leading to this expression are contained in the Supplementary Material section. It is easy to show that:


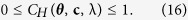


Notice that in order to evaluate (15) for a specific memory task, only Cov(*y*(*t*), **x**(*t*)) and var (*y*(*t*)) need to be computed since the autocovariance Γ(0) is fully determined by (14) once the reservoir and the equilibrium **x**_0_ around which we operate have been chosen. As an example, we provide the expressions corresponding to the two most basic information processing routines, namely the linear and the quadratic memory tasks. Details on how to obtain the following equalities are contained in the Supplementary Material section.

### The *h*-lag linear memory task

Linear memory tasks are those associated to linear task functions 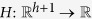, that is, if we denote 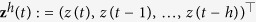 and 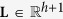, we set 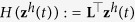. Various computations included in the Supplementary Material section using the so called MA(∞) representation of the VAR(1) process show that 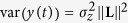, and 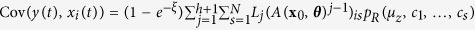, 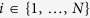, where the polynomial *p*_*R*_ on the variable *x* is defined by 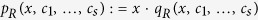 and its evaluation in the previous formula follows the same convention as in (11).

### The *h*-lag quadratic memory task

In this case we use a quadratic task function of the form


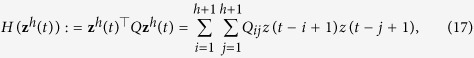


for some symmetric *h* + 1-dimensional matrix *Q*. If we define 
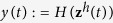
, we have that 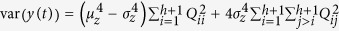, and


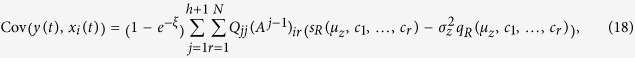


where the polynomial *s*_*R*_ on the variable *x* is defined as 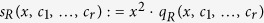.

## Discussion

The possibility to approximate the TDR using a model of the type (8) opens the door to the theoretical treatment of many RC design related questions that so far were addressed using a trial and error approach. In particular, the availability of a closed form formula of the type (15) for the memory capacity of the RC is extremely convenient to determine the optimal reservoir architecture to carry out a given task. Nevertheless, it is obviously very important to assess the quality of the VAR(1) approximation underlying it and of the consequences that result from it. Indeed, we recall that the expression (15) was obtained via the partial linearization of the reservoir at a stable equilibrium in which it is initialized and kept in stationary operation. Despite the good theoretical and experimental reasons to proceed in this fashion provided above, we have confirmed their pertinence by explicitly comparing the reservoir memory capacity surfaces obtained empirically with those coming from the analytical expression (15). We have carried this comparison out for various tasks and have constructed the memory capacity surfaces as a function of different design parameters.

**Figure 3:**
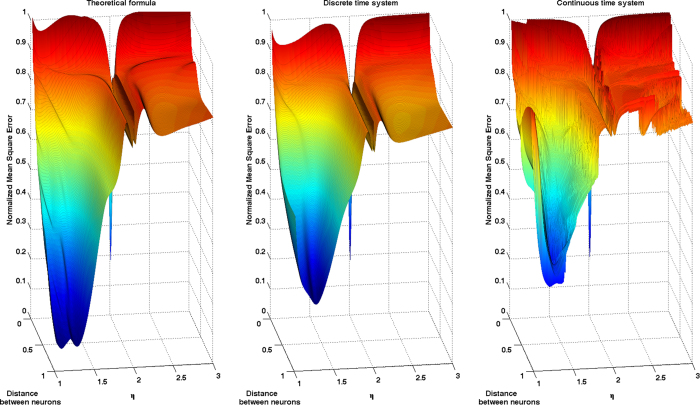
Error surfaces exhibited by a Mackey-Glass kernel based reservoir computer in a 6-lag quadratic memory task, as a function of the distance between neurons and the parameter *η*. The points in the surfaces of the middle and right panels are the result of Monte Carlo evaluations of the NMSE exhibited by the discrete and continuous time TDRs, respectively. The left panel was constructed using the formula (15) that is obtained as a result of modeling the reservoir with an approximating VAR(1) model. The computational convenience of the formula (15) can be visualized by noticing that each point in the middle and right panels took 37 and 41 seconds, respectively, to be estimated using a computer code written down in a high level programming language running on a single 2.53 GHz Intel i5 core; the same computation using (15) in the left panel took only 1.1 seconds.

We first consider a RC constructed using the Mackey-Glass nonlinear kernel (7) with *p* = 2, *γ* = 0.796, and twenty neurons. We present to it the 6-lag quadratic memory task *H* corresponding to choosing in (17) a seven dimensional diagonal matrix *Q* with the diagonal entries given by the vector (0, 1, 1, 1, 1, 1, 1). The first element, corresponding to the 0-lag memory (quadratic nowcasting), is set to zero in order to keep the difficulty of the task high enough. We then vary the value *d* of the distance between neurons between 0 and 1 and the feedback gain parameter *η* between 1 and 3. As we already discussed, the TDR in autonomous regime exhibits for these parameter values two stable equilibria placed at ±(*η* − 1)^1/2^; for this experiment we will always work with the positive equilibria by initializing the TDRs at those points. Fig. 3 represents the normalized mean square error (NMSE) surfaces (which amounts to one minus the capacity) obtained using three different approaches. The left panel was obtained using the formula (15) constructed with an eight-order Taylor expansion of the nonlinear kernel on the signal input (*R* = 8 in (9)). The points in the surfaces of the middle and right panels are the result of Monte Carlo evaluations (using 50,000 occurrences each) of the NMSE exhibited by the discrete and continuous time TDRs, respectively. The time-evolution of the time-delay differential equation (continuous time model) was simulated using a Runge-Kutta fourth-order method with a discretization step equal to *d*/5. A quick inspection of Fig. 3 reveals the ability of (15) to accurately capture most of the details of the error surface and, most importantly, the location in parameter space where optimal performance is attained; it is very easy to visualize in this particular example how sensitive the magnitude of the error and the corresponding memory capacity are to the choice of parameters and how small in size the region in parameter space associated with acceptable operation performance may be.

**Figure 4:**
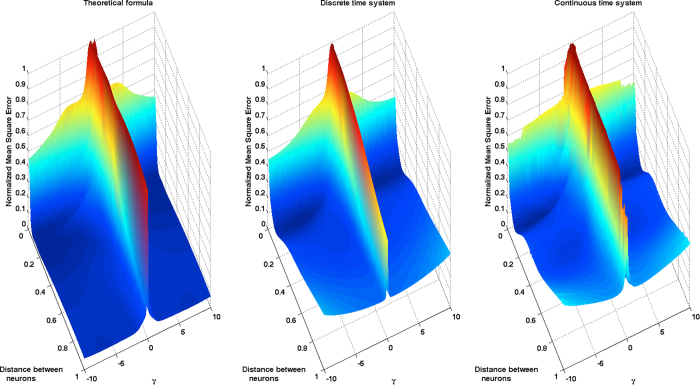
Error surfaces exhibited by a Mackey-Glass kernel based reservoir computer in a 3-lag quadratic memory task, as a function of the distance between neurons and the parameter *γ*. The points in the surfaces of the middle and right panels are the result of Monte Carlo evaluations of the NMSE exhibited by the discrete and continuous time TDRs, respectively. The left panel was constructed using the formula (15) that is obtained as a result of modeling the reservoir with an approximating VAR(1) model.

In order to show that these statements are robust with respect to the choice of task and varying parameters, we have carried out a similar experiment with a RC in which we fix the feedback gain *η*_0_ = 1.0781 and we vary the input gain *γ* and the distance between neurons *d*. The quadratic memory task is reduced this time to 3-lags. We emphasize that in this setup the stable operation point is always the same and equal to (*η*_0_ − 1)^1/2^. Fig. 4 shows how the performance of the memory capacity estimate (15) at the time of capturing the optimal parameter region is in this situation comparable to the results obtained for the 6-lag quadratic memory task represented in Fig. 3. We also point out that in this case there is a lower variability of the performance which, in our opinion, has to do with the fact that modifying the parameter *γ* adjusts the input gain but leaves unchanged the operation point. Additionally, the moderate difficulty of the task makes possible attaining lower optimal error rates with the same number of neurons. In order to ensure the robustness of these results with respect to the choice of nonlinear kernel, we have included in the Supplementary Material section the results of a similar experiment carried out using the Ikeda prescription.

**Figure 5:**
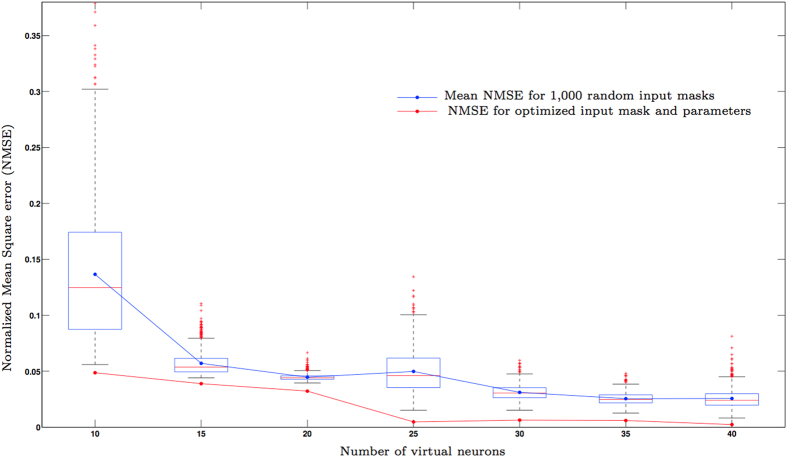
Influence of the mask optimization on the reservoir performance in the 3-lag quadratic memory task. The red line links the points that indicate the error committed by a RC with optimized parameters and mask. The box plots give information about the distribution of performances obtained with 1,000 input masks randomly picked (only reservoir parameters have been optimized). As it is customary, on each box, the central mark is the median and the edges of the box are the 25th and 75th percentiles (*q*_1_ and *q*_3_, respectively). The whiskers extend to the most extreme data points not considered outliers and outliers are plotted individually using red crosses. Points are drawn as outliers if they are larger than *q*_3_ + 1.5(*q*_3_ −*q*_1_) or smaller than *q*_1_ − 1.5(*q*_3_ − *q*_1_). The blue line links the points that indicate the mean NMSE committed when using the 1,000 different randomly picked masks.

Once the adequacy of the memory capacity evaluation formula (15) has been established, we can use this result to investigate the influence of other architecture parameters in the reservoir performance. In Fig. 5 we depict the results of an experiment where we study the influence of the choice of input mask **c** in the performance of a Mackey-Glass kernel based reservoir in a 3-lag quadratic memory task. The figure shows, for each number of neurons, the performance obtained by a RC in which the reservoir parameters ***θ*** and the input mask **c** have been chosen so that the memory capacity *C*_*H*_(***θ***, **c**, λ) in (15) is maximized; we have subsequently kept the optimal parameters ***θ*** and we have randomly constructed one thousand input masks **c** with entries belonging to the interval [−3, 3]. The box plots in Fig. 5 give an idea of the distribution of the degraded performances with respect to the optimal mask for different numbers of virtual neurons.

In conclusion, the construction of approximating models for the reservoir as well as the availability of performance evaluation formulas like (15) based on it, constitute extremely valuable analytical tools whose existence should prove very beneficial in the fast and efficient extension and customization of RC type techniques to tasks far more sophisticated than the ones we considered in this paper. This specific point is the subject of ongoing research on which we will report in a forthcoming publication.

## Additional Information

**How to cite this article**: Grigoryeva, L. *et al.* Optimal nonlinear information processing capacity in delay-based reservoir computers. *Sci. Rep.* **5**, 12858; doi: 10.1038/srep12858 (2015).

## Supplementary Material

Supplementary Information

## Figures and Tables

**Figure 1:**
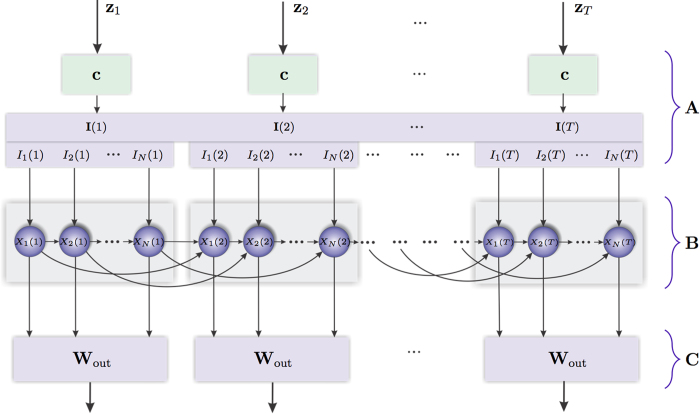
Neural diagram representing the architecture of the time-delay reservoir (TDR) and the three modules of the reservoir computer (RC): (**A**) is the input layer, (**B)** is the time-delay reservoir, and (**C**) is the readout layer.
