# Supplementary material for: Optimal nonlinear information processing capacity in delay-based reservoir computers
Source: Sci Rep. 2015 Sep 11;5:12858. doi: 10.1038/srep12858 (PMC4566100; doi:10.1038/srep12858)
Supplement: Supplementary Information [file srep12858-s1.pdf]

Supplementary material for the paper "Optimal nonlinear information processing capacity in delay-based reservoir computers"

Lyudmila Grigoryeva[1], Julie Henriques[1,2], Laurent Larger[3], and Juan-Pablo Ortega[4,*]

Abstract

This document contains technical appendices to the paper [1] that are meant for online publication, as a companion to the paper.

A Notation

Column vectors are denoted by bold lower or upper case symbol like $\mathbf{v}$ or $\mathbf{V}$. We write $\mathbf{v}^\top$ to indicate the transpose of $\mathbf{v}$. Given a vector $\mathbf{v} \in \mathbb{R}^n$, we denote its entries by v_i, with $i \in \{1, \ldots, n\}$; we also write $\mathbf{v} = (v_i)_{i\in\{1,\ldots,n\}}$. The symbols $\mathbf{i}_n$ and $\mathbf{0}_n$ stand for the vectors of length n consisting of zeros and ones, respectively.

We denote by $\mathbb{M}_{n,m}$ the space of real $n \times m$ matrices with $m, n \in \mathbb{N}$. When $n = m$, we use the symbol $\mathbb{M}_n$ to refer to the space of square matrices of order n. Given a matrix $A \in \mathbb{M}_{n,m}$, we denote its components by A_{ij} and we write $A = (A_{ij})$, with $i \in \{1, \ldots, n\}$, $j \in \{1, \ldots m\}$. We use $\mathbb{S}_n$ to indicate the subspace $\mathbb{S}_n \subset \mathbb{M}_n$ of symmetric matrices, that is, $\mathbb{S}_n = \left\{A \in \mathbb{M}_n \mid A^\top = A\right\}$. Given a matrix $A \in \mathbb{M}_{n,m}$, the maximum row sum matrix norm is defined as $|||A|||_\infty = \max_{1\leq i\leq n} \sum_{j=1}^m |A_{ij}|$. The symbol $\otimes$ stands for the Kronecker matrix product.

We use $C^r([a, b], \mathbb{R})$, $r \geq 0$, to denote the Banach space of the r-times continuously differentiable real valued maps defined on the interval $[a, b]$ with the topology of the uniform convergence; given a function $g \in C^r([a, b], \mathbb{R})$ when $r \geq 1$. We designate the l_∞-norm of an element $\phi \in C([a, b], \mathbb{R})$ by $||\phi||_\infty = \sup_{\theta\in[a,b]} |\phi(\theta)|$.

The symbols $\mathrm{E}[\cdot]$, $\mathrm{var}(\cdot)$, and $\mathrm{Cov}(\cdot, \cdot)$ denote the mathematical expectation, the variance, and the covariance, respectively.

[1]Laboratoire de Mathématiques de Besançon, UMR CNRS 6623, Université de Franche-Comté, UFR des Sciences et Techniques. 16, route de Gray. F-25030 Besançon cedex. France. `Lyudmyla.Grygoryeva@univ-fcomte.fr`

[2]Cegos Deployment. 11, rue Denis Papin. F-25000 Besançon. `jhenriques@deployment.org`

[3]FEMTO-ST, UMR CNRS 6174, Optics Department, Université de Franche-Comté, UFR des Sciences et Techniques. 15, Avenue des Montboucons. F-25000 Besançon cedex. France. `Laurent.Larger@univ-fcomte.fr`

[4]Corresponding author. Centre National de la Recherche Scientifique, Laboratoire de Mathématiques de Besançon, UMR CNRS 6623, Université de Franche-Comté, UFR des Sciences et Techniques. 16, route de Gray. F-25030 Besançon cedex. France. `Juan-Pablo.Ortega@univ-fcomte.fr`

B The reservoir map and the connectivity matrix

The reservoir map $F : \mathbb{R}^N \times \mathbb{R}^N \times \mathbb{R}^K \longrightarrow \mathbb{R}^N$ introduced in (4) is uniquely determined by the recursions (3) obtained out of the Euler discretization of the time-delay differential equation (TDDE)

$$\dot{x}(t) = -x(t) + f(x(t-\tau), I(t), \boldsymbol{\theta}), \tag{B.1}$$

and organized in neuron layers parametrized by $t \in \mathbb{Z}$. The reservoir map is obtained by using (3) in order to write down the neuron values of the layer for time t in terms of those for time $t-1$ and the current input signal value. More specifically:

$$\left\{ \begin{array}{rcl} x_1(t) &=& e^{-\xi} x_N(t-1) + (1-e^{-\xi}) f(x_1(t-1), I_1(t), \boldsymbol{\theta}), \\ x_2(t) &=& e^{-2\xi} x_N(t-1) + (1-e^{-\xi}) \left\{ e^{-\xi} f(x_1(t-1), I_1(t), \boldsymbol{\theta}) + f(x_2(t-1), I_2(t), \boldsymbol{\theta}) \right\}, \\ &\vdots& \\ x_N(t) &=& e^{-N\xi} x_N(t-1) + (1-e^{-\xi}) \sum_{j=0}^{N-1} e^{-j\xi} f(x_{N-j}(t-1), I_{N-j}(t), \boldsymbol{\theta}), \end{array} \right. \tag{B.2}$$

which corresponds to a description of the form

$$\mathbf{x}(t) = F(\mathbf{x}(t-1), \mathbf{I}(t), \boldsymbol{\theta}), \tag{B.3}$$

that uniquely determines the reservoir map $F : \mathbb{R}^N \times \mathbb{R}^N \times \mathbb{R}^K \longrightarrow \mathbb{R}^N$.

Let $x_0 \in \mathbb{R}$ and $\mathbf{x}_0 := x_0 \mathbf{i}_N \in \mathbb{R}^N$. Let $D_{\mathbf{x}} F(\mathbf{x}_0, \mathbf{0}_N, \boldsymbol{\theta})$ be the partial derivative of F with respect to the first argument computed at the point $(\mathbf{x}_0, \mathbf{0}_N, \boldsymbol{\theta})$. We will refer to $A(\mathbf{x}_0, \boldsymbol{\theta}) := D_{\mathbf{x}} F(\mathbf{x}_0, \mathbf{0}_N, \boldsymbol{\theta})$ as the **connectivity matrix** of the reservoir at the point $\mathbf{x}_0$. It is easy to show that $A(\mathbf{x}_0, \boldsymbol{\theta})$ has the following explicit form

$$A(\mathbf{x}_0, \boldsymbol{\theta}) := D_{\mathbf{x}} F(\mathbf{x}_0, \mathbf{0}_N, \boldsymbol{\theta}) = \begin{pmatrix} \Phi & 0 & \dots & 0 & e^{-\xi} \\ e^{-\xi}\Phi & \Phi & \dots & 0 & e^{-2\xi} \\ e^{-2\xi}\Phi & e^{-\xi}\Phi & \dots & 0 & e^{-3\xi} \\ \vdots & \vdots & \ddots & \vdots & \vdots \\ e^{-(N-1)\xi}\Phi & e^{-(N-2)\xi}\Phi & \dots & e^{-\xi}\Phi & \Phi + e^{-N\xi} \end{pmatrix}, \tag{B.4}$$

where $\Phi := (1-e^{-\xi}) \partial_x f(x_0, 0, \boldsymbol{\theta})$ and $\partial_x f(x_0, 0, \boldsymbol{\theta})$ is the first derivative of the nonlinear kernel f in (B.1) with respect to the first argument and computed at the point $(x_0, 0, \boldsymbol{\theta})$. We will also use the symbol f'_{x_0} to denote $\partial_x f(x_0, 0, \boldsymbol{\theta})$.

C The approximating vector autoregressive system and information processing capacity estimations

The goal of this appendix is providing details on the construction of the approximating VAR(1) model for the TDR obtained after a partial linearization of the reservoir map on the dynamical variables and respecting the nonlinearity on the input signal. Let $x_0 \in \mathbb{R}$ and $\mathbf{x}_0 := x_0 \mathbf{i}_N \in \mathbb{R}^N$ be an equilibrium of (B.1) and a fixed point of (B.3), respectively (see Proposition D.9). These solutions are chosen with respect to the autonomous regime, that is, we set $I(t) = 0$ in the time-delay differential equation (B.1) and $\mathbf{I}(t) = \mathbf{0}_N$ in the associated recursion (B.3). In practice, we choose stable solutions; Appendix D contains conditions that ensure this dynamical feature.

The VAR model setup and the construction of the approximating VAR system. The vector autoregressive (VAR) model is of much use in multivariate time series analysis and is a natural extension of the univariate linear autoregressive (AR) model. See [2] for an extensive introduction to multivariate

time series methods and the details on the VAR processes. The N-dimensional VAR(1) process (VAR model of order 1) is defined in mean-adjusted form as the solution to the recursions

$$\mathbf{x}(t) - \boldsymbol{\mu}_x = A(\mathbf{x}(t-1) - \boldsymbol{\mu}_x) + \boldsymbol{\epsilon}(t), \quad t = 0, \pm 1, \pm 2, \ldots \tag{C.1}$$

where $\mathbf{x}(t) = (x_1(t), \ldots, x_N(t))^\top \in \mathbb{R}^N$ is a random vector, $A \in \mathbb{M}_N$ is a fixed coefficient matrix, $\boldsymbol{\mu}_x \in \mathbb{R}^N$, and $\boldsymbol{\epsilon}(t) = (\epsilon_1(t), \ldots, \epsilon_N(t))^\top \in \mathbb{R}^N$ is such that $\{\boldsymbol{\epsilon}(t)\} \sim \mathrm{WN}(\mathbf{0}_N, \Sigma_\epsilon)$ is a N-dimensional white noise (stochastic process that presents no autocorrelation) or innovation process with mean $\mathbf{0}_N$ and covariance matrix $\Sigma_\epsilon \in \mathbb{S}_N$. We are particularly interested in **stable** VAR models, that is, models of the type (C.1) where the autoregression matrix A is chosen such that

$$\det\left(\mathbb{I}_N - Az\right) \neq 0 \quad \text{for all } z \in \mathbb{C} \text{ such that } |z| \leq 1. \tag{C.2}$$

It can be proved (see Proposition 2.1 in [2]) that stable models have a unique second order stationary solution $\{\mathbf{x}(t)\}_{t\in\mathbb{Z}}$ for which $\boldsymbol{\mu}_x = \mathrm{E}[\mathbf{x}(t)]$ and the autocovariance function

$$\Gamma(k) := \mathrm{E}\left[(\mathbf{x}(t) - \boldsymbol{\mu}_x)\,(\mathbf{x}(t-k) - \boldsymbol{\mu}_x)^\top\right], \quad k \in \mathbb{Z}$$

is time independent.

Let now $\mathbf{x}_0 = x_0 \mathbf{i}_N \in \mathbb{R}^N$ be the stable fixed point of the reservoir map (B.3) in autonomous regime, that is, $F(\mathbf{x}_0, \mathbf{0}_N, \boldsymbol{\theta}) = \mathbf{x}_0$. We now approximate (B.3) by its partial linearization at $\mathbf{x}_0$ with respect to the delayed self feedback and by the Rth-order Taylor series expansion on the variable that determines the input signal injection. We obtain the following expression:

$$\mathbf{x}(t) = F(\mathbf{x}_0, \mathbf{0}_N, \boldsymbol{\theta}) + D_{\mathbf{x}}F(\mathbf{x}_0, \mathbf{0}_N, \boldsymbol{\theta})(\mathbf{x}(t-1) - \mathbf{x}_0) + \boldsymbol{\varepsilon}(t), \tag{C.3}$$

where $D_{\mathbf{x}}F(\mathbf{x}_0, \mathbf{0}_N, \boldsymbol{\theta})$ is the first derivative of F with respect to its first argument, computed at the point $(\mathbf{x}_0, \mathbf{0}_N, \boldsymbol{\theta})$. We recall that $D_{\mathbf{x}}F(\mathbf{x}_0, \mathbf{0}_N, \boldsymbol{\theta}) = A(\mathbf{x}_0, \boldsymbol{\theta})$ is the connectivity matrix introduced in (B.4). Additionally, $\boldsymbol{\varepsilon}(t)$ in (C.3) is obtained out of the Taylor series expansion of $F(\mathbf{x}(t), \mathbf{I}(t), \boldsymbol{\theta})$ in (B.2) on $\mathbf{I}(t)$ up to some fixed order $R \in \mathbb{N}$ and is given by

$$\boldsymbol{\varepsilon}(t) = (1 - e^{-\xi})(q_R\left(z(t), c_1\right), q_R\left(z(t), c_1, c_2\right), \ldots, q_R\left(z(t), c_1, \ldots, c_N\right))^\top, \tag{C.4}$$

with

$$q_R\left(z(t), c_1, \ldots, c_r\right) := \sum_{i=1}^{R} \frac{z(t)^i}{i!} (\partial_I^{(i)} f)(x_0, 0, \boldsymbol{\theta}) \sum_{j=1}^{r} e^{-(r-j)\xi} c_j^i, \tag{C.5}$$

where c_i, $i \in \{1, \ldots, N\}$ are the entries of the input mask $\mathbf{c} \in \mathbb{R}^N$ and $(\partial_I^{(i)} f)(x_0, 0, \boldsymbol{\theta})$ is the ith order partial derivative of the nonlinear reservoir kernel f in (B.1) with respect to the second argument $I(t)$ computed at the point $(x_0, 0, \boldsymbol{\theta})$.

If we now use as input signal $z(t)$ independent and identically distributed random variables with mean 0 and variance σ_z^2, that is, $\{z(t)\}_{t\in\mathbb{Z}} \sim \mathrm{IID}(0, \sigma_z^2)$, then the recursion (C.3) makes the reservoir layer dynamics $\{\mathbf{x}(t)\}_{t\in\mathbb{Z}}$ into a discrete time random process that, as we show in what follows, is the solution of a N-dimensional VAR(1) model. Indeed, it is easy to see that the assumption $\{z(t)\}_{t\in\mathbb{Z}} \sim \mathrm{IID}(0, \sigma_z^2)$ implies that $\{\mathbf{I}(t)\}_{t\in\mathbb{Z}} \sim \mathrm{IID}(\mathbf{0}_N, \Sigma_I)$, with $\Sigma_I := \sigma_z^2 \mathbf{c}^\top \mathbf{c}$, and that $\{\boldsymbol{\varepsilon}(t)\}_{t\in\mathbb{Z}}$ is a family of N-dimensional independent and identically distributed random variables with mean $\boldsymbol{\mu}_\varepsilon$ and covariance matrix Σ_ε given by the following expression:

$$\boldsymbol{\mu}_\varepsilon = \mathrm{E}\left[\boldsymbol{\varepsilon}(t)\right] = (1 - e^{-\xi})\left(q_R\left(\mu_z, c_1\right), q_R\left(\mu_z, c_1, c_2\right), \ldots, q_R\left(\mu_z, c_1, \ldots, c_N\right)\right)^\top, \tag{C.6}$$

where the polynomial q_R is the same as in (C.5) and where we use the convention that the powers $\mu_z^i := \mathrm{E}\left[z(t)^i\right]$, for any $i \in \{1, \ldots, R\}$. For example, if the variables $z(t)$ are normal, that is, $\{z(t)\}_{t\in\mathbb{Z}} \sim \mathrm{IN}(0, \sigma_z^2)$, then

$$\mu_z^i := \mathrm{E}\left[z(t)^i\right] = \begin{cases} \dfrac{2l!}{2^l l!}\sigma_z^{2l} & \text{when } i = 2l, \quad l \in \mathbb{N}, \\ 0 & \text{otherwise.} \end{cases} \tag{C.7}$$

Additionally, $\Sigma_\varepsilon := \mathrm{E}\left[(\boldsymbol{\varepsilon}(t) - \boldsymbol{\mu}_\varepsilon)(\boldsymbol{\varepsilon}(t) - \boldsymbol{\mu}_\varepsilon)^\top\right]$ has entries determined by the relation:

$$(\Sigma_\varepsilon)_{ij} = (1 - e^{-\xi})^2((q_R(\cdot, c_1, \ldots, c_i) \cdot q_R(\cdot, c_1, \ldots, c_j))(\mu_z) - q_R(\mu_z, c_1, \ldots, c_i)q_R(\mu_z, c_1, \ldots, c_j)),$$

where the first summand stands for the multiplication of the polynomials $q_R(\cdot, c_1, \ldots, c_i)$ and $q_R(\cdot, c_1, \ldots, c_j)$ and the subsequent evaluation of the resulting polynomial at μ_z, and the second one is made out of the multiplication of the evaluation of the two polynomials.

With these observations it is clear that we can consider (C.3) as a VAR(1) model driven by the independent noise $\{\boldsymbol{\varepsilon}(t)\}_{t\in\mathbb{Z}}$. If the nonlinear kernel f satisfies the generic condition that the polynomial in z given by $\det(\mathbb{I}_N - A(\mathbf{x}_0, \boldsymbol{\theta})z)$, does not have roots in and on the complex unit circle, then (C.3) has a unique second order stationary solution $\{\mathbf{x}(t)\}_{t\in\mathbb{Z}}$ with time-independent mean

$$\boldsymbol{\mu}_x = \mathrm{E}\left[\mathbf{x}(t)\right] = (I_N - A(\mathbf{x}_0, \boldsymbol{\theta}))^{-1}(F(\mathbf{x}_0, \mathbf{0}_N, \boldsymbol{\theta}) - A(\mathbf{x}_0, \boldsymbol{\theta})\mathbf{x}_0 + \boldsymbol{\mu}_\varepsilon). \tag{C.8}$$

that can be used to rewrite (C.3) in mean-adjusted form

$$\mathbf{x}(t) - \boldsymbol{\mu}_x = A(\mathbf{x}_0, \boldsymbol{\theta})(\mathbf{x}(t-1) - \boldsymbol{\mu}_x) + (\boldsymbol{\varepsilon}(t) - \boldsymbol{\mu}_\varepsilon). \tag{C.9}$$

In the presence of stationarity we can recursively compute the time independent autocovariance function $\Gamma(k) := \mathrm{E}\left[(\mathbf{x}(t) - \boldsymbol{\mu}_x)(\mathbf{x}(t-k) - \boldsymbol{\mu}_x)^\top\right]$ at lag $k \in \mathbb{Z}$ by using the Yule-Walker equations [2]. Indeed, $\Gamma(0)$ is given by the vectorized equality:

$$\mathrm{vec}(\Gamma(0)) = (\mathbb{I}_{N^2} - A(\mathbf{x}_0, \boldsymbol{\theta}) \otimes A(\mathbf{x}_0, \boldsymbol{\theta}))^{-1} \mathrm{vec}(\Sigma_\varepsilon), \tag{C.10}$$

which determines the higher order autocovariances via the relation

$$\Gamma(k) = A(\mathbf{x}_0, \boldsymbol{\theta})\Gamma(k-1), \tag{C.11}$$

and the identity $\Gamma(-k) = \Gamma(k)^\top$.

The nonlinear memory capacity estimations. We now concentrate on the computation of the quantitative measures of the reservoir performance introduced in the paper. In particular, we will provide details on the computation of the nonlinear memory capacity formula in (13). Recall that a **h-lag memory task** is determined by a function $H : \mathbb{R}^{h+1} \to \mathbb{R}$ (in general nonlinear) that is used to generate a one-dimensional signal $y(t) := H(z(t), z(t-1), \ldots, z(t-h))$ out of the reservoir input $\{z(t)\}_{t\in\mathbb{Z}}$.

Consider now a TDR computer with N neurons. The optimal linear readout $\mathbf{W}_{\mathrm{out}}$ adapted to the memory task H is given by the solution of a ridge (or Tikhonov [3]) linear regression problem with regularization parameter $\lambda \in \mathbb{R}$ (usually tuned during the training phase via cross-validation) in which the covariates are the neuron values corresponding to the reservoir output and the explained variables are the values $\{y(t)\}$ of the memory task function. More explicitly, $\mathbf{W}_{\mathrm{out}}$ is given by the solution of the following optimization problem

$$(\mathbf{W}_{\mathrm{out}}, a_{\mathrm{out}}) := \underset{\mathbf{W}\in\mathbb{R}^N, a\in\mathbb{R}}{\arg\min} \left(\mathrm{E}\left[(\mathbf{W}^\top \cdot \mathbf{x}(t) + a - y(t))^2\right] + \lambda\|\mathbf{W}\|^2\right), \tag{C.12}$$

where the expectation is taken thinking of y_t and $\mathbf{x}(t)$ as random variables due to the stochastic nature of the input signal $\{z(t)\}_{t\in\mathbb{Z}}$ and hence that of the $\{\mathbf{I}(t)\}_{t\in\mathbb{Z}}$. In order to obtain the explicit solution of (C.12), we first define $g(\mathbf{W},a) := \mathrm{E}\left[(\mathbf{W}^\top \cdot \mathbf{x}(t) + a - y(t))^2\right] + \lambda\|\mathbf{W}\|^2$ and set

$$\begin{aligned}\frac{\partial g(\mathbf{W},a)}{\partial w_i} =& 2\left[\sum_{j=1}^{N} w_j \mathrm{E}\left[x_j(t)x_i(t)\right] + a\mathrm{E}\left[x_i(t)\right] - \mathrm{E}\left[y(t)x_i(t)\right] + \lambda w_i\right] = 0, \quad i \in \{1,\ldots,N\}\,,\\ \frac{\partial g(\mathbf{W},a)}{\partial a} =& 2\left[a + \mathbf{W}^\top \mathrm{E}\left[\mathbf{x}(t)\right] - \mathrm{E}\left[y(t)\right]\right] = 0,\end{aligned}$$

or, equivalently,

$$\begin{aligned}(\mathrm{Cov}(\mathbf{x}(t),\mathbf{x}(t)) + \lambda I_N)\mathbf{W} - \mathrm{Cov}(y(t),\mathbf{x}(t)) = 0,\\ a + \mathbf{W}^\top \mathrm{E}\left[\mathbf{x}(t)\right] - \mathrm{E}\left[y(t)\right] = 0.\end{aligned}$$

These equations yield the pair $(\mathbf{W}_{\mathrm{out}}, a_{\mathrm{out}})$ that minimizes $g(\mathbf{W},a)$. We now use the fact that $\{\mathbf{x}(t)\}_{t\in\mathbb{Z}}$ is the unique stationary solution of VAR(1) approximating system (C.9) for the TDR (C.9) and hence obtain

$$\begin{aligned}\mathbf{W}_{\mathrm{out}} =& (\Gamma(0) + \lambda I_N)^{-1}\mathrm{Cov}(y(t),\mathbf{x}(t)), && \text{(C.13)}\\ a_{\mathrm{out}} =& \mathrm{E}\left[y(t)\right] - \mathbf{W}_{\mathrm{out}}^\top \boldsymbol{\mu}_x, && \text{(C.14)}\end{aligned}$$

where $\boldsymbol{\mu}_x$ is provided in (C.8), $\Gamma(0) \in \mathbb{S}_N$ is determined by the generalized Yule-Walker equations in (C.10) and $\mathrm{Cov}(y(t),\mathbf{x}(t))$ is a vector in $\mathbb{R}^N$ that has to be determined for every specific memory task H. Additionally, it is easy to verify that the error committed by the reservoir when using the optimal readout is

$$\begin{aligned}&\mathrm{E}\left[\left(\mathbf{W}_{\mathrm{out}}^\top \cdot \mathbf{x}(t) + a_{\mathrm{out}} - y(t)\right)^2\right] = \mathbf{W}_{\mathrm{out}}^\top \Gamma(0)\mathbf{W}_{\mathrm{out}} + \mathrm{var}\left(y(t)\right) - 2\mathbf{W}_{\mathrm{out}}^\top \mathrm{Cov}(y(t),\mathbf{x}(t))\\ &= \mathrm{var}\left(y(t)\right) - \mathbf{W}_{\mathrm{out}}^\top(\Gamma(0) + 2\lambda\mathbb{I}_N)\mathbf{W}_{\mathrm{out}}\\ &= \mathrm{var}\left(y(t)\right) - \mathrm{Cov}(y(t),\mathbf{x}(t))^\top(\Gamma(0)+\lambda\mathbb{I}_N)^{-1}(\Gamma(0)+2\lambda\mathbb{I}_N)(\Gamma(0)+\lambda\mathbb{I}_N)^{-1}\mathrm{Cov}(y(t),\mathbf{x}(t)). \quad \text{(C.15)}\end{aligned}$$

The H-memory capacity $C_H(\boldsymbol{\theta},\mathbf{c},\lambda)$ of a reservoir computer constructed using a nonlinear kernel f with parameters $\boldsymbol{\theta}$, an input mask $\mathbf{c}$, and regularizing ridge parameter λ, is defined as one minus the normalized mean square error committed at the time of accomplishing the memory task H. Expression (C.15) shows that when the RC is approximated by the VAR(1) model (C.9), the corresponding **H-memory capacity** can be approximated by

$$C_H(\boldsymbol{\theta},\mathbf{c},\lambda) = \frac{\mathrm{Cov}(y(t),\mathbf{x}(t))^\top(\Gamma(0)+\lambda\mathbb{I}_N)^{-1}(\Gamma(0)+2\lambda\mathbb{I}_N)(\Gamma(0)+\lambda\mathbb{I}_N)^{-1}\mathrm{Cov}(y(t),\mathbf{x}(t))}{\mathrm{var}\left(y(t)\right)} \quad \text{(C.16)}$$

Since the normalized error coming from the expression (C.15) is clearly bounded between zero and one, it is also clear that:

$$0 \le C_H(\boldsymbol{\theta},\mathbf{c},\lambda) \le 1.$$

We emphasize that in order to evaluate (C.16) for a specific memory task, only $\mathrm{Cov}(y(t),\mathbf{x}(t))$ and $\mathrm{var}\left(y(t)\right)$ need to be computed since the autocovariance $\Gamma(0)$ is fully determined by (C.10) once the reservoir and the equilibrium $\mathbf{x}_0$ around which we operate have been chosen.

Once a specific reservoir and task H have been fixed, the capacity function $C_H(\boldsymbol{\theta},\mathbf{c},\lambda)$ can be explicitly written down and it can hence be used to find reservoir parameters $\boldsymbol{\theta}_{\mathrm{opt}}$ and an input mask $\mathbf{c}_{\mathrm{opt}}$ that maximize it, by solving the optimization problem

$$(\boldsymbol{\theta}_{\mathrm{opt}},\mathbf{c}_{\mathrm{opt}}) := \underset{\boldsymbol{\theta}\in\mathbb{R}^K,\mathbf{c}\in\mathbb{R}^N}{\arg\max}\; C_H(\boldsymbol{\theta},\mathbf{c},\lambda). \quad \text{(C.17)}$$

Two specific memory tasks. In the following paragraphs we spell out the computation of $\mathrm{Cov}(y(t), \mathbf{x}(t))$ and $\mathrm{var}\,(y(t))$ necessary to evaluate the memory capacity formula (C.16) for the two most basic memory tasks, namely, the linear and the quadratic ones.

(i) The h-lag linear memory task. The linear memory task is determined by the linear task functions $H : \mathbb{R}^{h+1} \to \mathbb{R}$ that we now describe. First, let $\mathbf{z}^h(t) := (z(t), z(t-1), \ldots, z(t-h))^\top$ and let $\mathbf{L} \in \mathbb{R}^{h+1}$. We then set $H(\mathbf{z}^h(t)) := \mathbf{L}^\top \mathbf{z}^h(t)$. In order to evaluate the h-lag memory capacity using formula (C.16), we need to evaluate $\mathrm{var}\,(y(t))$ and $\mathrm{Cov}(y(t), \mathbf{x}(t))$ with $y(t) := H(\mathbf{z}^h(t))$.

First, since $\{z(t)\}_{t\in\mathbb{Z}} \sim \mathrm{IID}(0, \sigma_z^2)$, we then immediately obtain that

$$\mathrm{var}(y(t)) = \sigma_z^2 ||\mathbf{L}||^2. \tag{C.18}$$

Next, we use the so called MA(∞)-representation of the VAR(1) in (C.9), namely,

$$(\mathbf{x}(t) - \boldsymbol{\mu}_x) = \sum_{i=0}^{\infty} \Psi_i \boldsymbol{\rho}(t-i), \tag{C.19}$$

with $\boldsymbol{\mu}_x$ as in (C.8), $\boldsymbol{\rho}(t) := \boldsymbol{\varepsilon}(t) - \boldsymbol{\mu}_\varepsilon$, $\boldsymbol{\mu}_\varepsilon$ defined in (C.6), $\Psi_i = A(\mathbf{x}_0, \boldsymbol{\theta})^i$, and $A(\mathbf{x}_0, \boldsymbol{\theta})$ the connectivity matrix of the discretized nonlinear TDR provided in (B.4). Using (C.19), we compute

$$\begin{aligned}
\mathrm{Cov}(y(t), x_i(t)) &= \mathrm{Cov}(\mathbf{L}^\top \mathbf{z}^h(t), x_i(t)) = \sum_{j=1}^{h+1} L_j \mathrm{Cov}(z(t-j+1), x_i(t)) \\
&= \sum_{j=1}^{h+1} \sum_{k=0}^{\infty} \sum_{r=1}^{N} L_j (A(\mathbf{x}_0, \boldsymbol{\theta})^k)_{ir} \mathrm{E}\left[z(t-j+1)\rho_r(t-k)\right] \\
&= \sum_{j=1}^{h+1} \sum_{r=1}^{N} L_j (A(\mathbf{x}_0, \boldsymbol{\theta})^{j-1})_{ir} \mathrm{E}\left[z(t)(\varepsilon_r(t) - z(t)(\boldsymbol{\mu}_\varepsilon)_r\right], \quad \text{with } i \in \{1, \ldots, N\},
\end{aligned} \tag{C.20}$$

which immediately yields that

$$\mathrm{Cov}(y(t), x_i(t)) = (1 - e^{-\xi}) \sum_{j=1}^{h+1} \sum_{r=1}^{N} L_j (A(\mathbf{x}_0, \boldsymbol{\theta})^{j-1})_{ir} p_R(\mu_z, c_1, \ldots, c_r), \quad \text{with } i \in \{1, \ldots, N\}, \tag{C.21}$$

where the polynomial p_R on the variable x is defined by $p_R(x, c_1, \ldots, c_r) := x \cdot q_R(x, c_1, \ldots, c_r)$ and its evaluation follows the same convention as in (C.6). The expressions (C.18) and (C.21) can be readily substituted in (C.16) in order to obtain an explicit expression for capacity $C_H(\boldsymbol{\theta}, \mathbf{c}, \lambda)$ associated to the h-lag linear memory task as a function of the reservoir parameters $\boldsymbol{\theta}$ and the input mask $\mathbf{c}$. This expression can be subsequently treated as in (C.17) in order to determine optimal architecture parameters for this particular task.

(ii) The h-lag quadratic memory task. In this case we use a quadratic task function $H : \mathbb{R}^{h+1} \to \mathbb{R}$ of the form

$$H(\mathbf{z}^h(t)) := \mathbf{z}^h(t)^\top Q \mathbf{z}^h(t) = \sum_{i=1}^{h+1} \sum_{j=1}^{h+1} Q_{ij} z(t-i+1) z(t-j+1), \tag{C.22}$$

for some symmetric matrix $Q \in \mathbb{S}_{h+1}$. Analogously to the linear task case, in order to evaluate the memory capacity associated to H, we have to derive explicit expressions for $\mathrm{var}\,(y(t))$ and

$\mathrm{Cov}(y(t), \mathbf{x}(t))$ with $y(t) := H(\mathbf{z}^h(t))$. The same computations as in the case of the linear task apply. First, if $\{z(t)\}_{t\in\mathbb{Z}} \sim \mathrm{IID}(0, \sigma_z^2)$, we can immediately write

$$\mathrm{E}\left[y(t)\right] = \sigma_z^2 \mathrm{tr}(Q), \tag{C.23}$$

and

$$\begin{aligned}
\mathrm{E}\left[y(t)^2\right] &= \sum_{i=1}^{h+1}\sum_{j=1}^{h+1}\sum_{k=1}^{h+1}\sum_{l=1}^{h+1} \mathrm{E}\left[Q_{ij}Q_{kl} z(t-i+1)z(t-j+1)z(t-k+1)z(t-l+1)\right] \\
&= \sum_{i=1}^{h+1} Q_{ii}^2 \mathrm{E}\left[z(t-i+1)^4\right] + 4\sum_{i=1}^{h+1}\sum_{j>i}^{h+1} Q_{ij}^2 \mathrm{E}\left[z(t-i+1)^2 z(t-j+1)^2\right] \\
&\quad +2\sum_{i=1}^{h+1}\sum_{j>i}^{h+1} Q_{ii}Q_{jj} \mathrm{E}\left[z(t-i+1)^2 z(t-j+1)^2\right] \\
&= \mu_z^4 \sum_{i=1}^{h+1} Q_{ii}^2 + 4\sigma_z^4 \sum_{i=1}^{h+1}\sum_{j>i}^{h+1} Q_{ij}^2 + 2\sigma_z^4 \sum_{i=1}^{h+1}\sum_{j>i}^{h+1} Q_{ii}Q_{jj}. \qquad (C.24) \\
& \qquad (C.25)
\end{aligned}$$

Analogously, by (C.23),

$$\mathrm{E}\left[y(t)\right]^2 = \sigma_z^4 \mathrm{tr}(Q)^2 = \sigma_z^4 \left(\sum_{i=1}^{h+1} Q_{ii}^2 + 2\sum_{i=1}^{h+1}\sum_{j>i}^{h+1} Q_{ii}Q_{jj} \right). \tag{C.26}$$

Hence, if we put together (C.24) and (C.26), we obtain

$$\mathrm{var}(y(t)) = \mathrm{E}\left[y(t)^2\right] - \mathrm{E}\left[y(t)\right]^2 = (\mu_z^4 - \sigma_z^4)\sum_{i=1}^{h+1} Q_{ii}^2 + 4\sigma_z^4 \sum_{i=1}^{h+1}\sum_{j>i}^{h+1} Q_{ij}^2. \tag{C.27}$$

Recall that for Gaussian variables, that is $\{z(t)\}_{t\in\mathbb{Z}} \sim \mathrm{IN}(0, \sigma_z^2)$, we have that $\mu_z^4 = 3\sigma_z^2$, and hence in that case

$$\mathrm{var}(y(t)) = 2\sigma_z^4 \left(\sum_{i=1}^{h+1} Q_{ii}^2 + 2\sum_{i=1}^{h+1}\sum_{j>i}^{h+1} Q_{ij}^2 \right) = 2\sigma_z^4 \sum_{i=1}^{h+1}\sum_{j=1}^{h+1} Q_{ij}^2. \tag{C.28}$$

Regarding the computation of the covariance and analogously to the case of the linear h-lag memory task, we use the $\mathrm{MA}(\infty)$ representation of the VAR(1) model of the TDR in (C.9) and write

$$\begin{aligned}
\mathrm{Cov}(y(t), x_i(t)) &= \sum_{j=1}^{h+1}\sum_{k=1}^{h+1}\sum_{l=1}^{\infty} Q_{jk} \mathrm{E}\left[z(t-j+1)z(t-k+1)(A(\mathbf{x}_0, \boldsymbol{\theta})^l \boldsymbol{\rho}(t-l))_i\right] \\
&= \sum_{l=0}^{\infty}\sum_{j=1}^{h+1}\sum_{k=1}^{h+1}\sum_{r=1}^{N} Q_{jk} (A(\mathbf{x}_0, \boldsymbol{\theta})^l)_{ir} \Big\{ \mathrm{E}\left[z(t-j+1)z(t-k+1)\varepsilon_r(t-l)\right] \\
&\quad - (\boldsymbol{\mu}_\varepsilon)_r \mathrm{E}\left[z(t-j+1)z(t-k+1)\right] \Big\}, \text{ with } i \in \{1, \ldots, N\},
\end{aligned}$$

which leads to the following result:

$$\mathrm{Cov}(y(t), x_i(t)) = (1 - e^{-\xi}) \sum_{j=1}^{h+1} \sum_{r=1}^{N} Q_{jj} (A(\mathbf{x}_0, \boldsymbol{\theta})^{j-1})_{ir} (s_R(\mu_z, c_1, \ldots, c_r) - \sigma_z^2 q_R(\mu_z, c_1, \ldots, c_r)), \tag{C.29}$$

with $i \in \{1, \ldots, N\}$. In this relation the polynomial s_R on x is defined as $s_R(x, c_1, \ldots, c_r) := x^2 \cdot q_R(x, c_1, \ldots, c_r)$ and is evaluated following the same convention as in (C.6) but taking x^2 instead of x.

Again, we conclude by noticing that the expressions (C.29) and (C.27) substituted in (C.16) provide an explicit formula for capacity $C_H(\boldsymbol{\theta}, \mathbf{c}, \lambda)$ associated to the h-lag quadratic memory task as a function of the reservoir parameters $\boldsymbol{\theta}$ and the input mask $\mathbf{c}$ and hence it can be readily used to solve the optimization problem in (C.17).

An observation that is worth to be pointed out is that only the diagonal elements in Q intervene in the covariance (C.29) while all its entries are present in the variance (C.27). When these two quantities are substituted in the memory capacity formula (C.16) it can be seen that by choosing sufficiently high off-diagonal entries in Q, the capacity of the reservoir can be made arbitrarily small which shows a structural limitation of the architecture that we are considering that can only be fixed by using alternative signal feeding schemes.

D Equilibria of the continuous and the discrete time models for the TDR and their stability

As we already explained, the linearization of the reservoir map at a stable fixed point is at the core of the developments in this paper. That is why in this section we carry out a detailed study of the stability properties of the equilibria of the time-delay differential equation (B.1) and of the fixed points of its corresponding discrete-time approximation (B.3). More specifically, we provide sufficient stability conditions and we show that our results exhibit a remarkable consistence regardless of the use of the continuous or of the discrete time schemes.

D.1 Stationary solutions of time-delay differential equations and their stability

We start by recalling some basic facts about the properties of the solutions of the time-delay differential equations and their stability. Let $\tau \in \mathbb{R}^+$ be a fixed delay and consider a **time-delay map**

$$\begin{array}{rccc} X: & C^1([-\tau, 0], \mathbb{R}) \times \mathbb{R} & \longrightarrow & \mathbb{R} \\ & (\gamma, t) & \longmapsto & X(\gamma, t). \end{array} \tag{D.1}$$

Additionally, for any $t \in \mathbb{R}$ define the **shift operator**

$$\begin{array}{rccc} S_t: & C^1([-\tau + t, t], \mathbb{R}) & \longrightarrow & C^1([-\tau, 0], \mathbb{R}) \\ & \gamma & \longmapsto & \gamma \circ \lambda_t, \end{array} \tag{D.2}$$

where λ_t is the translation operator by $t \in \mathbb{R}$, that is, $\lambda_t(s) := s + t$, for any $s \in \mathbb{R}$. Let now $\gamma \in C^1([-\tau, +\infty), \mathbb{R})$ be a differentiable curve. We say that γ is a solution of the **time-delay differential equation (TDDE) determined by** X when the equality

$$\dot{\gamma}(t) = X(S_t \circ \gamma|_{[-\tau+t,t]}, t) \tag{D.3}$$

holds for any $t \in [0, +\infty)$. Note that the TDDE (B.1) that is at the core of this paper, namely

$$\dot{x}(t) = -x(t) + f(x(t-\tau), I(t), \boldsymbol{\theta}), \tag{D.4}$$

can be encoded as in (D.3) by using the time-delay map X given by

$$\begin{array}{rccc} X: & C^1([-\tau,0],\mathbb{R}) \times \mathbb{R} & \longrightarrow & \mathbb{R} \\ & (\gamma, t) & \longmapsto & -\gamma(0) + f(\gamma(-\tau), I(t), \boldsymbol{\theta}). \end{array} \tag{D.5}$$

Definition D.1 *We say that the time-delay map X is locally Lipschitzian on the open set $\Omega \subset C^1([-\tau,0],\mathbb{R}) \times \mathbb{R}$ if it is Lipschitzian in any compact subset of Ω, that is, for any compact subset Ω_0 of Ω there exists a constant $K \in \mathbb{R}^+$ such that for all (γ_1, t) and (γ_2, t) in Ω_0 one has*

$$|X(\gamma_1, t) - X(\gamma_2, t)| < K||\gamma_1 - \gamma_2||_\infty. \tag{D.6}$$

Theorem D.2 (Existence and uniqueness of solutions) *Let X be a continuous and locally Lipschitzian time-delay map in $C^1([-\tau,0],\mathbb{R}) \times \mathbb{R}$. Then, for any $\phi \in C^1([-\tau,0],\mathbb{R})$ there exists a unique $\Gamma_\phi \in C^1([-\tau,+\infty),\mathbb{R})$ such that*

$$\begin{cases} \Gamma_\phi(t) & = \phi(t), & \text{for any } t \in [-\tau, 0] \\ \dot{\Gamma}_\phi(t) & = X(S_t \circ \Gamma_\phi|_{[-\tau+t,t]}, t), & \text{for any } t \in (0, +\infty]. \end{cases} \tag{D.7}$$

We say that Γ_ϕ is the **solution** *of the time-delay differential equation determined by X with initial condition ϕ, or simply the solution through ϕ. The associated* **flow** *is defined as the map*

$$\begin{array}{rccc} F: & [-\tau,+\infty) \times C^1([-\tau,0],\mathbb{R}) & \longrightarrow & \mathbb{R} \\ & (t, \phi) & \longmapsto & \Gamma_\phi(t) \end{array} \tag{D.8}$$

and note that $F.(\phi) \in C^1([-\tau,+\infty),\mathbb{R})$.

We now recall also some basic notions of stability of common use in the TDDE context; see [4] and [5] for details. Let $x_0 \in \mathbb{R}$ and let $\phi_{x_0} \in C^1([-\tau,0],\mathbb{R})$ be the constant curve at x_0. We say that the point x_0 is an **equilibrium** of the TDDE determined by the time-delay map and with flow F whenever $F_t(\phi_{x_0}) = x_0$, for any $t \in [-\tau, +\infty)$. The equilibrium x_0 is said to be **stable** (respectively **asymptotically stable**) if for any $\epsilon > 0$ there exists a $\delta(\epsilon) > 0$ such that for any $\phi \in C^1([-\tau,0],\mathbb{R})$ with $\|\phi - \phi_{x_0}\|_\infty < \delta(\epsilon)$, we have that $|F_t(\phi) - x_0| < \epsilon$, for any $t \in [-\tau, +\infty)$ (respectively $\lim_{t\to\infty} F_t(\phi) = x_0$).

The following stability criterion is an extension of Lyapunov's Second Method to the TDDE context due to Krasovskiy [6]. We state it using our notation since it will be used in the sequel.

Theorem D.3 (Lyapunov-Krasovskiy stability theorem) *let $x_0 \in \mathbb{R}$ be an equilibrium of the time-delay differential equation (D.3) with flow $F : [-\tau,+\infty) \times C^1([-\tau,0],\mathbb{R})) \longrightarrow \mathbb{R}$. Let u, v, $w : \overline{\mathbb{R}^+} \longrightarrow \overline{\mathbb{R}^+}$ be continuous nondecreasing functions such that $u(0) = v(0) = 0$ and $u(t), v(t), w(t) > 0$ for any $t \in (0, +\infty)$. If there exists a continuously differentiable functional V*

$$V: \quad C^1([-\tau,+\infty),\mathbb{R}) \times \mathbb{R} \longrightarrow \mathbb{R} \tag{D.9}$$

such that for any $\phi \in C^1([-\tau,0],\mathbb{R}))$ and any $t \in [0,+\infty)$ satisfies that

(i) $u(|\phi(0)|) \leq V(F.(\phi), t) \leq v(||\phi||_\infty)$,

(ii) $\dot{V}(F.(\phi), t) := \dfrac{d}{dt} V(F.(\phi), t) \leq -w(|\phi(0)|)$,

then x_0 is asymptotically stable. If $w(t) \geq 0$ then x_0 is just stable. A functional V that satisfies these conditions is called a **Lyapunov-Krasovskiy functional**.

D.2 Equilibria of the reservoir time-delay equation and their stability

We now use Theorem D.3 to establish sufficient conditions for the stability of the equilibria of the TDDE (B.1) at the core of the paper, namely,

$$\dot{x}(t) = x(t) + f(x(t-\tau), I(t), \boldsymbol{\theta}). \tag{D.10}$$

where f is the nonlinear kernel of the TDR. The main tool in the application of that result is the use of a Lyapunov-Krasovskiy functional of the form

$$\begin{array}{rccc} V: & C^1([-\tau,+\infty],\mathbb{R})\times\mathbb{R} & \longrightarrow & \mathbb{R} \\ & (x_\phi, t) & \longmapsto & \dfrac{1}{2}x_\phi(t)^2 + \mu\int_{t-\tau}^{t} x_\phi(s)^2 ds, \end{array} \tag{D.11}$$

where $\mu \in \mathbb{R}^+$ and $x_\phi = F.(\phi)$ for some initial curve $\phi \in C^1([-\tau, 0], \mathbb{R})$. See [6], [4] and [5] for the extensive discussion.

Theorem D.4 *Let x_0 be an equilibrium of the time-delay differential equation (D.10) in autonomous regime, that is, when $I(t) = 0$, and suppose that there exists $\varepsilon > 0$ and $k_\varepsilon \in \mathbb{R}$ such that one of the following conditions holds*

(i) $f(x + x_0, 0, \boldsymbol{\theta}) \leq k_\varepsilon x + x_0$ *for all* $x \in (-\varepsilon, \varepsilon)$

(ii) $\dfrac{f(x + x_0, 0, \boldsymbol{\theta}) - x_0}{x} \leq k_\varepsilon$ *for all* $x \in (-\varepsilon, \varepsilon)$.

If $|k_\varepsilon| < 1$ then x_0 is **asymptotically stable**. *If $|k_\varepsilon| \leq 1$ then x_0 is* **stable**.

Proof. Notice first that the equilibria x_0 in the statement are characterized by the equality $f(x_0, 0, \boldsymbol{\theta}) = x_0$. Consider now the Lyapunov-Krasovskiy functional introduced in (D.11). It is easy to see that since $V(x_\phi, t)$ is positive it satisfies condition **(i)** in Theorem D.9. We will now show that any of the two conditions in the statement imply that condition **(ii)** in Theorem D.9 are satisfied and hence guarantee the stability of x_0. We start by writing

$$\begin{aligned} \frac{d}{dt}V(x_\phi, t) &= x_\phi(t)\dot{x}_\phi(t) + \mu\left(x_\phi(t)^2 - x_\phi(t-\tau)^2\right) \\ &= -x_\phi(t)^2 + x_\phi(t) f(x_\phi(t-\tau), 0, \boldsymbol{\theta}) + \mu\left(x_\phi(t)^2 - x_\phi(t-\tau)^2\right). \end{aligned} \tag{D.12}$$

We now distinguish two cases, namely, when $x_0 = 0$ and when $x_0 \neq 0$.

Case $x_0 = 0$. Suppose that $x_0 = 0$ is a solution of the TDDE (D.10). Under the hypothesis **(i)** in the statement, in the case of the trivial solution $x_0 = 0$ there exists $\varepsilon > 0$ and $k_\varepsilon > 0$ such that $f(x, 0, \boldsymbol{\theta}) \leq k_\varepsilon x$ for all $x \in (-\varepsilon, \varepsilon)$, and hence from (D.12) we can conclude that

$$\begin{aligned} \frac{d}{dt}V(x_\phi, t) &\leq -x_\phi(t)^2 + k_\varepsilon x_\phi(t) x_\phi(t-\tau) + \mu\left(x_\phi(t)^2 - x_\phi(t-\tau)^2\right) \\ &= \left(x_\phi(t), x_\phi(t-\tau)\right) Q \left(x_\phi(t), x_\phi(t-\tau)\right)^\top \end{aligned} \tag{D.13}$$

with

$$Q := \begin{pmatrix} \mu - 1 & k_\varepsilon/2 \\ k_\varepsilon/2 & -\mu \end{pmatrix}.$$

Expression (D.13) is negative for any $(x_\phi(t), x_\phi(t-\tau))$ if the matrix Q is negative definite which by the Sylvester's law amounts to $\mu < 1$ and $k_\varepsilon^2 < -4\mu(\mu-1)$. Since $-4\mu(\mu-1)$ has a maximum at $\mu = 1/2$ for which $-4\mu(\mu-1) = 1$, we obtain from Theorem D.3 that the optimal sufficient condition for asymptotic

stability of x_0 is $|k_\varepsilon| < 1$ as required. Analogously, by Theorem D.3, a sufficient condition for $x_0 = 0$ to be stable is the non-positivity of expression (D.13) or, equivalently, the negative semi-definiteness of Q which amounts to $\mu \le 1$ and $k_\varepsilon^2 \le -4\mu(\mu - 1)$. Hence the optimal sufficient condition for the stability of $x_0 = 0$ is $|k_\varepsilon| \le 1$ as required.

Consider (D.12) again, now under the hypothesis **(ii)** of the statement of the theorem. In the case of the trivial solution it implies that there exists $\varepsilon > 0$ and $k_\varepsilon > 0$, such that $\frac{f(x, 0, \boldsymbol{\theta})}{x} \le k_\varepsilon$ for all $x \in (-\varepsilon, \varepsilon)$. In order to ensure the asymptotic stability of the trivial solution using Theorem D.3, we need to find conditions under which the expression (D.12) is negative, that is $\frac{d}{dt}V(x_\phi, t) < 0$. We proceed by first multiplying both sides of this inequality by the positive quantity $\frac{1}{x_\phi(t-\tau)^2}$. We obtain

$$-\frac{x_\phi(t)^2}{x_\phi(t-\tau)^2} + \frac{x_\phi(t) f(x_\phi(t-\tau), 0, \boldsymbol{\theta})}{x_\phi(t-\tau)^2} + \mu\left(\frac{x_\phi(t)^2}{x_\phi(t-\tau)^2} - 1\right) < 0.$$

Then due to the hypothesis **(ii)** of the theorem, a sufficient condition for this inequality to hold is

$$-\frac{x_\phi(t)^2}{x_\phi(t-\tau)^2} + k_\varepsilon \frac{x_\phi(t)}{x_\phi(t-\tau)} + \mu\left(\frac{x_\phi(t)^2}{x_\phi(t-\tau)^2} - 1\right) < 0. \tag{D.14}$$

Notice that when $x_\phi(t) = x_\phi(t-\tau) = x_0$, this inequality is always satisfied provided that $k_\varepsilon \le 1$. Hence in order for (D.14) to hold, it suffices that the polynomial on z

$$-z^2 + k_\varepsilon z + \mu(z^2 - 1) = (\mu - 1)z^2 + k_\varepsilon z - \mu \tag{D.15}$$

has no real roots, which happens, as in point **(i)** of the statement when $k_\varepsilon^2 < -4\mu(\mu - 1)$. Proceeding analogously as under assumption **(i)** of the theorem, we obtain $|k_\varepsilon| < 1$ (respectively $|k_\varepsilon| \le 1$) as the sufficient condition for the asymptotic stability (respectively stability) of $x_0 = 0$, as required.

Case $x_0 \neq 0$. Suppose now that $x_0 \neq 0$ and define the new variable $y(t) := x(t) - x_0$. With this change of variables the equation (D.10) becomes

$$\dot{y}(t) = \dot{x}(t) = -x_0 - y(t) + f(y(t-\tau), 0, \boldsymbol{\theta})$$

or, equivalently,

$$\dot{y}(t) = -y(t) + g(y(t-\tau), 0, \boldsymbol{\theta}), \tag{D.16}$$

where the function g is defined as $g(y(t), 0, \boldsymbol{\theta}) := f(y(t) + x_0, 0, \boldsymbol{\theta}) - x_0$. The equation (D.2) has an equilibrium at $y_0 = 0$ whose stability can be easily studied by mimicking the case $x_0 = 0$ discussed above. More specifically, it can be shown following the same arguments that in this case the hypothesis **(i)** of the statement of the theorem can be written as

$$g(y, 0, \boldsymbol{\theta}) \le k_\varepsilon y \tag{D.17}$$

and $y_0 = 0$ is stable or asymptotically stable whenever $|k_\varepsilon| \le 1$ or $|k_\varepsilon| < 1$, respectively. The inequality (D.17) is equivalent to

$$f(y + x_0, 0, \boldsymbol{\theta}) - x_0 \le k_\varepsilon y$$

or

$$f(y + x_0, 0, \boldsymbol{\theta}) \le k_\varepsilon y + x_0,$$

which guarantees that a non-trivial equilibrium x_0 of (D.10) is stable or asymptotically stable when the same conditions on k_ε as in the trivial case are satisfied.

Finally, the hypothesis **(ii)** of the statement of the theorem in the case of (D.16) has the form

$$\frac{g(y,0,\boldsymbol{\theta})}{y} \leq k_\varepsilon \tag{D.18}$$

and $y_0 = 0$ is stable or asymptotically stable when $|k_\varepsilon| \leq 1$ or $|k_\varepsilon| < 1$, respectively. It is easy to verify that the inequality (D.18) is equivalent to

$$\frac{f(y+x_0,0,\boldsymbol{\theta}) - x_0}{y} \leq k_\varepsilon, \tag{D.19}$$

which provides the same corresponding sufficient conditions on k_ε for stability or asymptotic stability of a non-trivial equilibrium x_0 of (D.10), as required. □

Corollary D.5 *Let x_0 be an equilibrium of the TDDE (D.10) and suppose that the nonlinear reservoir kernel function f is continuously differentiable at x_0. If $|\partial_x f(x_0,0,\boldsymbol{\theta})| < 1$ (respectively, $|\partial_x f(x_0,0,\boldsymbol{\theta})| \leq 1$), then x_0 is asymptotically stable (respectively, stable).*

Proof. First, define the function

$$g_\varepsilon(h) := \begin{cases} \dfrac{f(x_0+h,0,\boldsymbol{\theta}) - f(x_0,0,\boldsymbol{\theta})}{h}, & h \neq 0, h \in (-\varepsilon,\varepsilon) \quad \text{(D.20a)} \\ \partial_x f(x_0,0,\boldsymbol{\theta}) = \lim\limits_{h\to 0} \dfrac{f(x_0+h,0,\boldsymbol{\theta}) - f(x_0,0,\boldsymbol{\theta})}{h}, & h = 0. \quad \text{(D.20b)} \end{cases}$$

By construction, the function g_ε is continuous in $(-\varepsilon,\varepsilon)$, that is $g_\varepsilon \in C^0((-\varepsilon,\varepsilon),\mathbb{R})$. Hence, by the Weierstrass extreme value theorem, this function reaches a maximum k_ε in the interval $[-\varepsilon/2,\varepsilon/2]$, that is,

$$g_\varepsilon(h) = \frac{f(x_0+h,0,\boldsymbol{\theta}) - f(x_0,0,\boldsymbol{\theta})}{h} \leq k_\varepsilon \quad \text{for any} \quad h \in [-\varepsilon/2,\varepsilon/2]. \tag{D.21}$$

Since x_0 is an equilibrium, then $f(x_0,0,\boldsymbol{\theta}) = x_0$ and the condition (D.21) coincides with the hypothesis **(ii)** of Theorem D.4. The equilibrium x_0 can be hence proved to be asymptotically stable (respectively, stable) if $|k_\varepsilon| < 1$ (respectively, $|k_\varepsilon| \leq 1$). Additionally, using (D.20a)-(D.20b) and the continuity of g_ε, it is easy to see that

$$\lim_{\varepsilon\to 0} k_\varepsilon = \lim_{\varepsilon\to 0} g_\varepsilon(h) = \partial_x f(x_0,0,\boldsymbol{\theta})$$

and hence the asymptotic stability (respectively, stability) of x_0 is guaranteed if $|\partial_x f(x_0,0,\boldsymbol{\theta}| < 1$ (respectively, $|\partial_x f(x_0,0,\boldsymbol{\theta}| \leq 1$), as required. □

We now study the equilibria and the parameter values that ensure their stability when Corollary D.5 is applied to the two nonlinear kernels that are most used in our work, that is, the Mackey-Glass [7] and the Ikeda [8] parametric families. We recall that the **Mackey-Glass nonlinear kernel** is given by the expression

$$f(x,I,\boldsymbol{\theta}) = \frac{\eta\,(x+\gamma I)}{1+(x+\gamma I)^p}, \tag{D.22}$$

where the parameter $\boldsymbol{\theta} := (\gamma,\eta,p) \in \mathbb{R}^3$ is a three tuple of real values. The **Ikeda nonlinear kernel** corresponds to

$$f(x,I,\boldsymbol{\theta}) = \eta\sin^2(x+\gamma I+\phi), \tag{D.23}$$

where the parameter vector $\boldsymbol{\theta} := (\gamma,\eta,\phi) \in \mathbb{R}^3$. In both cases the parameter γ is called the **input gain** and η the **feedback gain**.

Corollary D.6 (Stability of the equilibria of the Mackey-Glass TDDE) *Consider the TDDE (D.10) in the autonomous regime constructed with the Mackey-Glass kernel (D.22) with $p = 2$, that is,*

$$f(x, 0, \boldsymbol{\theta}) = \frac{\eta x}{1 + x^2}. \tag{D.24}$$

This TDDE exhibits two families of equilibria depending on the values of η:

(i) *The trivial solution $x_0 = 0$, for any $\eta \in \mathbb{R}$. The equilibrium $x_0 = 0$ is asymptotically stable (respectively, stable) if $|\eta| < 1$ (respectively, $|\eta| \leq 1$).*

(ii) *The non-trivial solutions $x_0 = \pm\sqrt{\eta - 1}$, for any $\eta > 1$. The equilibria $x_0 = \pm\sqrt{\eta - 1}$ are asymptotically stable (respectively, stable) whenever $1 < \eta < 3$ (respectively, $1 < \eta \leq 3$).*

Proof. First, in order to characterize the equilibria of the time-delay differential equation (D.10) with the nonlinear kernel in (D.24), we solve $0 = -x + f(x, 0, \boldsymbol{\theta})$ or, equivalently,

$$\frac{\eta x}{1 + x^2} - x = 0.$$

A straightforward computation shows that this equality is equivalent to $x(x^2 - (\eta - 1)) = 0$ which immediately yields the two families of equilibria in the statement, namely, $x_0 = 0$, $\forall \eta \in \mathbb{R}$ and $x_0 = \pm\sqrt{\eta - 1}$ for any $\eta > 1$. We now use Corollary D.5 of Theorem D.4, in order to provide the sufficient conditions for stability and asymptotic stability of these two families. Using (D.24), we obtain that

$$\partial_x f(x, 0, \boldsymbol{\theta}) = \frac{\eta(1 - x^2)}{1 + x^2}. \tag{D.25}$$

Then, when we evaluate this expression at the equilibria under study, we obtain:

(i) for $x_0 = 0$, we have that $\partial_x f(x_0, 0, \boldsymbol{\theta}) = \eta$ and hence by Corollary D.5 the trivial solution x_0 is asymptotically stable (respectively, stable) if $|\eta| < 1$ (respectively, $|\eta| \leq 1$).

(ii) for $x_0 = \pm\sqrt{\eta - 1}$ with $\eta > 1$ the expression (D.25) amounts to $\partial_x f(x_0, 0, \boldsymbol{\theta}) = 2 - \eta$ and hence by Corollary D.5 the non-trivial solutions x_0 are asymptotically stable (respectively, stable) whenever $\eta \in (1, 3)$ (respectively, $\eta \in (1, 3]$), as required.

□

Corollary D.7 (Stability of the equilibria of the Ikeda TDDE) *Consider the TDDE (D.10) in the autonomous regime constructed with the Ikeda kernel (D.23), that is,*

$$f(x, 0, \boldsymbol{\theta}) = \eta \sin^2(x + \phi). \tag{D.26}$$

The Ikeda nonlinear TDDE exhibits two families of equilibria:

(i) *The trivial solution $x_0 = 0$ for any $\eta \in \mathbb{R}$ and $\phi = \pi n$, $n \in \mathbb{Z}$. The equilibium $x_0 = 0$ is asymptotically stable for any $\eta \in \mathbb{R}$.*

(ii) *The non-trivial equilibria x_0 are obtained as solutions of the equation $x_0 = \eta \sin^2(x_0 + \phi)$, for any $\eta \in \mathbb{R}$ and $\phi \neq \pi n$, $n \in \mathbb{Z}$. These equilibria are asymptotically stable (respectively, stable) whenever*

$$|\sin(2x_0 + 2\phi)| < \frac{1}{|\eta|} \quad (\textit{respectively, } |\sin(2x_0 + 2\phi)| \leq \frac{1}{|\eta|}). \tag{D.27}$$

When $|\eta| < 1$ (respectively, $|\eta| \leq 1$), there exists only one non-trivial equilibrium that is always asymptotically stable (respectively, stable).

Proof. The equilibria of the time-delay differential equation (D.10) with the Ikeda kernel (D.26), are characterized by the roots x_0 of the equation $0 = -x + f(x, 0, \boldsymbol{\theta})$ or, equivalently,

$$\eta \sin^2(x + \phi) - x = 0. \tag{D.28}$$

We divide the solutions of this equation into two families, namely, the trivial equilibrium $x_0 = 0$, for any $\eta \in \mathbb{R}$ and $\phi = \pi n$, $n \in \mathbb{Z}$, and the non-trivial ones obtained when $\phi \neq \pi n$, $n \in \mathbb{Z}$. Using (D.26), we compute

$$\partial_x f(x, 0, \boldsymbol{\theta}) = \eta \sin(2x + 2\phi) \tag{D.29}$$

and evaluate it at the two families of equilibria under study.

(i) For $x_0 = 0$ the expression (D.29) yields $\partial_x f(x_0, 0, \boldsymbol{\theta}) = \eta \sin(2\phi) \equiv 0$, since $\phi = \pi n$, $n \in \mathbb{Z}$. Hence by Corollary D.5 the trivial solution x_0 is always asymptotically stable.

(ii) For non-trivial equilibria x_0, the expression (D.29) amounts to $\partial_x f(x_0, 0, \boldsymbol{\theta}) = \eta \sin(2x_0 + 2\phi)$ and hence by Corollary D.5 the non-trivial solutions x_0 are asymptotically stable (respectively, stable) whenever $|\sin(2x_0 + 2\phi)| < \frac{1}{|\eta|}$ (respectively, $|\sin(2x_0 + 2\phi)| \leq \frac{1}{|\eta|}$). We now consider the case $|\eta| < 1$ (respectively, $|\eta| \leq 1$); in that situation the stability inequalities (D.27) always hold true but it remains to be shown that only one equilibrium exists. That claim is a consequence of the following lemma.

Lemma D.8 *If $|\eta| < 1$, then the equation (D.28) has at most one root.*

Proof of Lemma. Consider the function $g(x) := \eta \sin^2(x + \phi) - x$. As $g'(x) = \eta \sin(2x + 2\phi) - 1$, we have that if $|\eta| \leq 1$, then $g'(x) \leq \eta - 1 \leq 0$ for any $x \in \mathbb{R}$. The function $g(x)$ is hence a monotonously decreasing function and intersects the OX axis in at most one point. Since $g(0) > 0$ (recall that in this case $\phi \neq \pi n$, $n \in \mathbb{Z}$) and for any $x > \eta$ we have that $g(x) < 0$, we conclude that $g(x)$ intersects the OX axis in exactly one point, as required. □

Figure 1 illustrates the statement of Corollary D.7.

D.3 Fixed points of the reservoir map and their stability

In this section we consider the discrete time TDR, we characterize its fixed points and establish sufficient conditions for their stability which, as we will show, are analogous to the ones that we obtained for the continuous time case.

We place ourselves in the autonomous regime, that is, $I(t) = 0$ in the time-delay differential equation (B.1) and $\mathbf{I}(t) = \mathbf{0}_N$ in the associated recursion (B.3). In this case, we can state the following proposition that shows that there is a bijective correspondence between the equilibria of the discrete and continuous time TDRs.

Proposition D.9 *The point $x_0 \in \mathbb{R}$ is an equilibrium of the time-delay differential equation (D.10) in autonomous regime, that is when $I(t) = 0$, if and only if the vector $\mathbf{x}_0 := x_0 \mathbf{i}_N$ is a fixed point of the N-dimensional discretized nonlinear time-delay reservoir*

$$\dot{\mathbf{x}}(t) = F(\mathbf{x}(t-1), \mathbf{I}(t), \boldsymbol{\theta}) \tag{D.30}$$

in autonomous regime, that is, when $\mathbf{I}(t) = \mathbf{0}_N$.

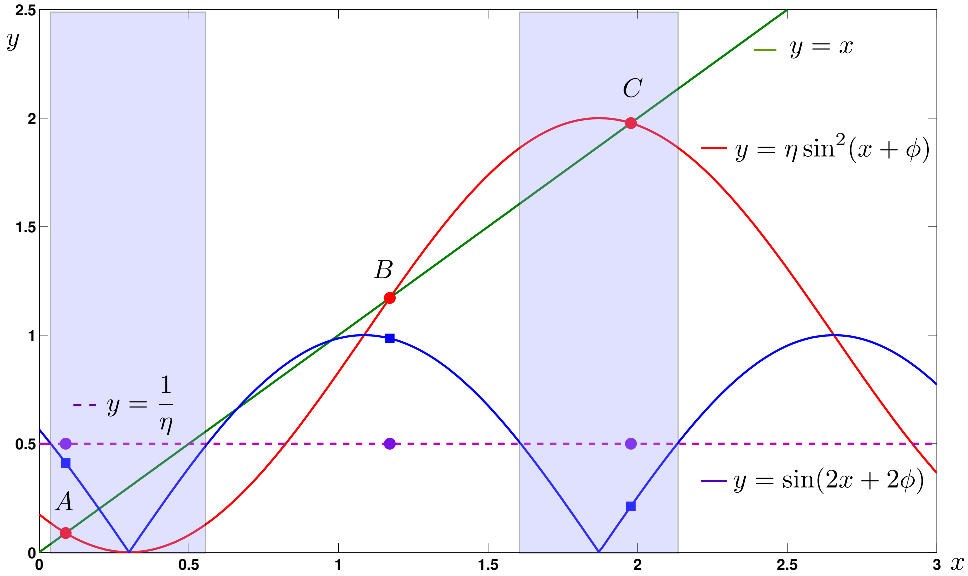

Figure 1: Illustration of the statement of Corollary D.7. The parameter vector $\boldsymbol{\theta}$ in (D.26) is set to $(2, -0.3)$. Non-trivial equilibria $x_0 = 0.088$ (point A), $x_0 = 1.172$ (point B), $x_0 = 1.977$ (point C) are provided by the relation (D.28). Grey bands represent the regions where the sufficient stability conditions (D.27) in the statement of the corollary are satisfied; the equilibria that correspond to the points A and C are hence stable.

Proof. Suppose first that x_0 is an equilibrium of the time-delay differential equation (D.10) and hence satisfies $x_0 = f(x_0, 0, \boldsymbol{\theta})$. In order to show that $F(\mathbf{x}(t-1), \mathbf{0}_N, \boldsymbol{\theta}) = \mathbf{x}_0$, we evaluate the components of the right hand side of (B.2) at $\mathbf{x}_0$ and obtain

$$(F(\mathbf{x}_0, \mathbf{0}_N, \boldsymbol{\theta}))_i = e^{-i\xi}x_0 + (1 - e^{-\xi}) \sum_{j=0}^{i-1} e^{-j\xi} f(x_0, 0, \boldsymbol{\theta}) = e^{-i\xi}x_0 + (1 - e^{-\xi}) \sum_{j=0}^{i-1} e^{-j\xi} x_0 = x_0,$$

as required. The proof of the converse implication is also straightforward using (B.2). $\square$

We now provide sufficient conditions for the stability of the fixed points of the type $\mathbf{x}_0 = x_0 \mathbf{i}_N$ described in Proposition D.9. The asymptotic stability (respectively, stability) of those fixed points is guaranteed whenever the connectivity matrix $A(\mathbf{x}_0, \boldsymbol{\theta})$ in (B.4) satisfies $\rho(A(\mathbf{x}_0, \boldsymbol{\theta})) < 1$ (respectively, $\rho(A(\mathbf{x}_0, \boldsymbol{\theta})) \leq 1$)). Since it is not possible to compute the eigenvalues of $A(\mathbf{x}_0, \boldsymbol{\theta})$ for an arbitrary number of neurons N, we proceed by finding upper bounds of its spectral radius $\rho(A(\mathbf{x}_0, \boldsymbol{\theta}))$. This can be done with the help of the Gershgorin disks theorem (see for instance Corollary 6.1.5 in [9]) or by using a matrix norm $|||\cdot|||$ and noting that $\rho(A(\mathbf{x}_0, \boldsymbol{\theta})) \leq |||A(\mathbf{x}_0, \boldsymbol{\theta})|||$. After a detailed study using all these possibilities we found that the best result is obtained by using the maximum row sum matrix norm $|||A(\mathbf{x}_0, \boldsymbol{\theta})|||_\infty$ defined in Section A, which allows us to formulate the following result.

Theorem D.10 *Let $\mathbf{x}_0 = x_0 \mathbf{i}_N$ be a fixed point of the N-dimensional recursion $\mathbf{x}(t) = F(\mathbf{x}(t-1), \mathbf{I}(t), \boldsymbol{\theta})$ in autonomous regime. Then, $\mathbf{x}_0 \in \mathbb{R}^N$ is asymptotically stable (respectively stable) if $|\partial_x f(x_0, 0, \boldsymbol{\theta})| < 1$ (respectively, $|\partial_x f(x_0, 0, \boldsymbol{\theta})| \leq 1$).*

Proof. We first recall that the connectivity matrix (B.4) of the discretized nonlinear TDR with N

virtual nodes is given by

$$A(\mathbf{x}_0,\boldsymbol{\theta}) := \begin{pmatrix} \Phi & 0 & \dots & 0 & e^{-\xi} \\ \Phi e^{-\xi} & \Phi & \dots & 0 & e^{-2\xi} \\ \Phi e^{-2\xi} & \Phi e^{-\xi} & \dots & 0 & e^{-3\xi} \\ \vdots & \vdots & \vdots & & \vdots \\ \Phi e^{-(N-1)\xi} & \Phi e^{-(N-2)\xi} & \dots & \Phi e^{-\xi} & \Phi + e^{-N\xi} \end{pmatrix}, \tag{D.31}$$

where $\Phi := (1-e^{-\xi}) \cdot \partial_x f(x_0, 0, \boldsymbol{\theta})$ and recall that $\partial_x f(x_0, 0, \boldsymbol{\theta})$ is the first derivative of the nonlinear kernel f in (B.1) with respect to the first argument and computed at the point $(\mathbf{x}_0, \mathbf{0}_N, \boldsymbol{\theta})$, with $\xi = \log(1+d)$ and $d \in (0,1]$ the Euler discretization step or, equivalently, the separation between the virtual neurons. We will use the notation $f'_{x_0} := \partial_x f(x_0, 0, \boldsymbol{\theta})$ in what follows.

We proceed by finding sufficient conditions on f'_{x_0} that guarantee that the spectral radius of $A(\mathbf{x}_0, \boldsymbol{\theta})$ is bounded above by 1. These conditions will be obtained by enforcing $|||A(\mathbf{x}_0,\boldsymbol{\theta})|||_\infty < 1$ and by recalling that

$$\rho(A(\mathbf{x}_0,\boldsymbol{\theta})) \leq |||A(\mathbf{x}_0,\boldsymbol{\theta})|||_\infty = \max_{1\leq i\leq N} \sum_{j=1}^{N} |a_{ij}|. \tag{D.32}$$

In the view of the rows of the matrix $A(\mathbf{x}_0,\boldsymbol{\theta})$, it is clear that $|||A(\mathbf{x}_0,\boldsymbol{\theta})|||_\infty$ is given by the sum of the absolute values of one of the rows with numbers 1, $N-1$, or N. We can hence write

$$|||A(\mathbf{x}_0,\boldsymbol{\theta})|||_\infty = \max \left\{ \begin{array}{ll} u := & e^{-\xi} + (1-e^{-\xi})|f'_{x_0}| \\ v := & e^{-(N-1)\xi} + (1-e^{-(N-1)\xi})|f'_{x_0}| \\ w := & |e^{-N\xi} + (1-e^{-\xi})f'_{x_0}| + e^{-\xi}(1-e^{-(N-1)\xi})|f'_{x_0}| \end{array} \right\}. \tag{D.33}$$

It can be easily verified that this expression can be split into two cases, namely

$$|||A(\mathbf{x}_0,\boldsymbol{\theta})|||_\infty = \begin{cases} \max\{u,w\}, & \text{if } |f'_{x_0}| \leq 1 \quad \text{(D.34a)} \\ \max\{v,w\}, & \text{if } |f'_{x_0}| \geq 1 \quad \text{(D.34b)} \end{cases}$$

Additionally, by definition of the absolute value, we have two cases:

$$|f'_{x_0}| = \begin{cases} f'_{x_0}, & \text{if } f'_{x_0} \in [0,+\infty) \quad \text{(D.35a)} \\ -f'_{x_0}, & \text{if } f'_{x_0} \in (-\infty, 0) \quad \text{(D.35b)} \end{cases}$$

and hence

$$|e^{-N\xi} + (1-e^{-\xi})f'_{x_0}| = \begin{cases} e^{-N\xi} + (1-e^{-\xi})f'_{x_0}, & \text{if } f'_{x_0} \in \left[-\dfrac{e^{-N\xi}}{1-e^{-\xi}}, +\infty\right) \quad \text{(D.36a)} \\ -e^{-N\xi} - (1-e^{-\xi})f'_{x_0}, & \text{if } f'_{x_0} \in \left(-\infty, -\dfrac{e^{-N\xi}}{1-e^{-\xi}}\right). \quad \text{(D.36b)} \end{cases}$$

We now consider in detail all the possible combinations of cases that provide the conditions on f'_{x_0} that ensure stability by enforcing that $|||A(\mathbf{x}_0,\boldsymbol{\theta})|||_\infty < 1$.

Case I (D.34a), (D.35a), (D.36a).

On one hand, (D.34a), (D.35a), (D.36a) give that $f'_{x_0} \in [0,1)$. On the other hand (D.34a) amounts to

$$|||A(\mathbf{x}_0,\boldsymbol{\theta})|||_\infty = \max\{u,w\} = \begin{cases} u, & \text{if } f'_{x_0} \in (-\infty, 1] \quad \text{(D.37a)} \\ w, & \text{if } f'_{x_0} \in (1,+\infty). \quad \text{(D.37b)} \end{cases}$$

Notice that since $f'_{x_0} \in [0,1)$, then these cases reduce to:

$$|||A(\mathbf{x}_0,\boldsymbol{\theta})|||_\infty = u, \tag{D.38}$$

and hence

$$\rho(A(\mathbf{x}_0,\boldsymbol{\theta})) \le u = e^{-\xi} + (1-e^{-\xi})f'_{x_0} < 1 \implies f'_{x_0} \in (-\infty, 1).$$

We finally write that in this case stability is guaranteed whenever

$$f'_{x_0} \in I1 := [0,1). \tag{D.39}$$

Case II (D.34a)**,** (D.35a)**,** (D.36b)**.**
On one hand, (D.34a), (D.35a) imply that $f'_{x_0} \in [0,1)$ but by (D.36b) it is required at the same time that $f'_{x_0} \in (-\infty, -\frac{e^{-N\xi}}{1-e^{-\xi}})$ which immediately yields in this case:

$$f'_{x_0} \in I2 := \emptyset. \tag{D.40}$$

Case III (D.34a)**,** (D.35b)**,** (D.36a)**.**
On one hand, (D.34a), (D.35b), (D.36a) imply that

$$f'_{x_0} \in \Big[\max\Big\{-1, -\frac{e^{-N\xi}}{1-e^{-\xi}}\Big\}, 0\Big). \tag{D.41}$$

On the other hand, the condition that defines (D.34a) amounts to

$$|||A(\mathbf{x}_0,\boldsymbol{\theta})|||_\infty = \max\{u,w\} = \begin{cases} u, & \text{if } f'_{x_0} \in \left[-\frac{e^{-\xi}-e^{-N\xi}}{|2-3e^{-\xi}+e^{-N\xi}|}, \frac{e^{-\xi}-e^{-N\xi}}{|2-3e^{-\xi}+e^{-N\xi}|}\right] \quad \text{(D.42a)} \\ w, & \text{if } f'_{x_0} \in \left(-\infty, -\frac{e^{-\xi}-e^{-N\xi}}{|2-3e^{-\xi}+e^{-N\xi}|}\right) \cup \left(\frac{e^{-\xi}-e^{-N\xi}}{|2-3e^{-\xi}+e^{-N\xi}|}, +\infty\right) \quad \text{(D.42b)} \end{cases}.$$

Notice now that at the same time we require that $|||A(\mathbf{x}_0,\boldsymbol{\theta})|||_\infty < 1$. Hence for the case (D.42a) we have

$$\rho(A(\mathbf{x}_0,\boldsymbol{\theta})) \le |||A(\mathbf{x}_0,\boldsymbol{\theta})|||_\infty = u = e^{-\xi} - (1-e^{-\xi})f'_{x_0} < 1 \Longrightarrow f'_{x_0} \in (-1,+\infty) \tag{D.43}$$

which put together with the conditions for f'_{x_0} in (D.42a), (D.43), and (D.41) yields

$$f'_{x_0} \in I3 := \left(\max\left\{-1, -\frac{e^{-N\xi}}{1-e^{-\xi}}, -\frac{e^{-\xi}-e^{-N\xi}}{|2-3e^{-\xi}+e^{-N\xi}|}\right\}, 0\right). \tag{D.44}$$

We now consider the case (D.42b) and in an analogous way using (D.3) we have that

$$\rho(A(\mathbf{x}_0,\boldsymbol{\theta})) \le |||A(\mathbf{x}_0,\boldsymbol{\theta})|||_\infty = w = e^{-N\xi} + (1-2e^{-\xi}+e^{-N\xi})f'_{x_0} < 1 \Longrightarrow$$
$$f'_{x_0} \in \left(-\frac{1-e^{-N\xi}}{|1-2e^{-\xi}+e^{-N\xi}|}, \frac{1-e^{-N\xi}}{|1-2e^{-\xi}+e^{-N\xi}|}\right) \tag{D.45}$$

If we put together the conditions for f'_{x_0} in (D.42b), (D.45), and (D.41) we obtain

$$f'_{x_0} \in I4 := \left(\max\left\{-1, -\frac{e^{-N\xi}}{1-e^{-\xi}}, -\frac{1-e^{-N\xi}}{|1-2e^{-\xi}+e^{-N\xi}|}\right\}, -\frac{e^{-\xi}-e^{-N\xi}}{|2-3e^{-\xi}+e^{-N\xi}|}\right). \tag{D.46}$$

Case IV (D.34a)**,** (D.35b)**,** (D.36b)**.**

On one hand, (D.34a), (D.35b), (D.36b) imply that

$$f'_{x_0} \in \Big(-1, -\frac{e^{-N\xi}}{1-e^{-\xi}} \Big). \tag{D.47}$$

On the other hand, the case (D.34a) amounts to

$$|||A(\mathbf{x}_0,\boldsymbol{\theta})|||_\infty = \max\{u,w\} = \begin{cases} u, & \text{if } f'_{x_0} \in \Big[-\frac{1+e^{-(N-1)\xi}}{1-e^{-(N-1)\xi}}, +\infty \Big) & \text{(D.48a)} \\ w, & \text{if } f'_{x_0} \in \Big(-\infty, -\frac{1+e^{-(N-1)\xi}}{1-e^{-(N-1)\xi}} \Big). & \text{(D.48b)} \end{cases}$$

It can be easily verified that the condition defining (D.48b) is incompatible with (D.47) since the relation $-1 \geq -\frac{1+e^{-(N-1)\xi}}{1-e^{-(N-1)\xi}}$ holds for any $\xi \in (0,1]$ and $N \in \mathbb{N}$. We hence conclude using the case (D.48a) that

$$\rho(A(\mathbf{x}_0,\boldsymbol{\theta})) \leq |||A(\mathbf{x}_0,\boldsymbol{\theta})|||_\infty = u = e^{-\xi} - (1-e^{-\xi})f'_{x_0} < 1 \Longrightarrow f'_{x_0} \in (-1,+\infty).$$

Hence, together with (D.47) this amounts to

$$f'_{x_0} \in I5 := \Big(-1, -\frac{e^{-N\xi}}{1-e^{-\xi}} \Big). \tag{D.49}$$

Case V (D.34b), (D.35a), (D.36a).
On one hand, (D.34b), (D.35a), (D.36a) imply that

$$f'_{x_0} \in [1,+\infty]. \tag{D.50}$$

On the other hand, the case (D.34a) amounts to

$$|||A(\mathbf{x}_0,\boldsymbol{\theta})|||_\infty = \max\{v,w\} = \begin{cases} v, & \text{if } f'_{x_0} \in (-\infty,-1] & \text{(D.51a)} \\ w, & \text{if } f'_{x_0} \in (-1,+\infty). & \text{(D.51b)} \end{cases}$$

Due to (D.50)

$$\rho(A(\mathbf{x}_0,\boldsymbol{\theta})) \leq |||A(\mathbf{x}_0,\boldsymbol{\theta})|||_\infty = w = e^{-N\xi} + (1-e^{-N\xi})f'_{x_0} < 1 \Longrightarrow f'_{x_0} \in (-\infty,1),$$

which yields

$$f'_{x_0} \in I6 := \emptyset. \tag{D.52}$$

Case VI (D.34b), (D.35a), (D.36b).
The conditions (D.34b), (D.35a), and (D.36b) immediately yield

$$f'_{x_0} \in I7 := \emptyset. \tag{D.53}$$

Case VII (D.34b), (D.35b), (D.36a).
On one hand, (D.34b), (D.35b), (D.36a) imply that

$$f'_{x_0} \in \Big[-\frac{e^{-N\xi}}{1-e^{-\xi}}, -1 \Big]. \tag{D.54}$$

On the other hand, the case (D.34b) amounts to

$$|||A(\mathbf{x}_0,\boldsymbol{\theta})|||_\infty = \max\{v,w\} = \begin{cases} v, & \text{if } f'_{x_0} \in \Big(-\infty, -\frac{e^{-(N-1)\xi}}{2-e^{-(N-1)\xi}} \Big], & \text{(D.55a)} \\ w, & \text{if } f'_{x_0} \in \Big(-\frac{e^{-(N-1)\xi}}{2-e^{-(N-1)\xi}}, +\infty \Big). & \text{(D.55b)} \end{cases}$$

Hence, for the case (D.55a) we have

$$\rho(A(\mathbf{x}_0,\boldsymbol{\theta})) \leq |||A(\mathbf{x}_0,\boldsymbol{\theta})|||_\infty = v = e^{-(N-1)\xi} - (1-e^{-(N-1)\xi})f'_{x_0} < 1 \Longrightarrow f'_{x_0} \in (-1,+\infty)$$

which due to (D.54) gives

$$f'_{x_0} \in I8 := \emptyset. \tag{D.56}$$

We now consider the case (D.55b) and in an analogous way we have that

$$\rho(A(\mathbf{x}_0,\boldsymbol{\theta})) \leq |||A(\mathbf{x}_0,\boldsymbol{\theta})|||_\infty = w = e^{-N\xi} + (1-2e^{-\xi}+e^{-N\xi})f'_{x_0} < 1 \Longrightarrow$$
$$f'_{x_0} \in \left(-\frac{1-e^{-N\xi}}{|1-2e^{-\xi}+e^{-N\xi}|}, \frac{1-e^{-N\xi}}{|1-2e^{-\xi}+e^{-N\xi}|}\right). \tag{D.57}$$

Hence by (D.57), (D.55b) and (D.54) we can write

$$f'_{x_0} \in I9 := \left(\max\left\{-\frac{e^{-N\xi}}{1-e^{-\xi}}, -\frac{e^{-(N-1)\xi}}{2-e^{-(N-1)\xi}}, -\frac{1-e^{-N\xi}}{|1-2e^{-\xi}+e^{-N\xi}|}\right\}, -1\right] = \emptyset, \tag{D.58}$$

where the last equality follows from the fact that $-\frac{e^{-(N-1)\xi}}{2-e^{-(N-1)\xi}} \geq -1$, for any $\xi \in (0,1]$ and $N \in \mathbb{N}$.

Case VIII (D.34b), (D.35b), (D.36b).

On one hand, (D.34b), (D.35b), (D.36b) imply that

$$f'_{x_0} \in \left(-\infty, \min\left\{-\frac{e^{-N\xi}}{1-e^{-\xi}}, -1\right\}\right). \tag{D.59}$$

On the other hand, the case (D.34b) amounts to

$$|||A|||_\infty = \max\{v,w\} = \begin{cases} v, & \text{if } f'_{x_0} \in \left[-\frac{1+e^{-\xi}}{1-e^{-\xi}}, +\infty\right) & \text{(D.60a)} \\ w, & \text{if } f'_{x_0} \in \left(-\infty, -\frac{1+e^{-\xi}}{1-e^{-\xi}}\right). & \text{(D.60b)} \end{cases}$$

Hence for the case (D.60a) we have

$$\rho(A(\mathbf{x}_0,\boldsymbol{\theta})) \leq |||A(\mathbf{x}_0,\boldsymbol{\theta})|||_\infty = v = e^{-(N-1)\xi} - (1-e^{-(N-1)\xi})f'_{x_0} < 1 \Longrightarrow f'_{x_0} \in (-1,+\infty)$$

which due to (D.59) implies that

$$f'_{x_0} \in I10 := \emptyset. \tag{D.61}$$

We now consider the case (D.60b) and in an analogous way we have that

$$\rho(A(\mathbf{x}_0,\boldsymbol{\theta})) \leq |||A(\mathbf{x}_0,\boldsymbol{\theta})|||_\infty = w = -e^{-N\xi} + (-1+e^{-N\xi})f'_{x_0} < 1 \Longrightarrow f'_{x_0} \in \left(-\frac{1+e^{-N\xi}}{1-e^{-N\xi}}, +\infty\right). \tag{D.62}$$

Hence by (D.62), (D.60b) and (D.59) we can write

$$f'_{x_0} \in I11 := \left(-\frac{1+e^{-N\xi}}{1-e^{-N\xi}}, \min\left\{-\frac{e^{-N\xi}}{1-e^{-\xi}}, -1, -\frac{1+e^{-\xi}}{1-e^{-\xi}}\right\}\right) = \emptyset, \tag{D.63}$$

for any $\xi \in (0,1]$ and $N \in \mathbb{N}$.

Finally, we put together all the non-empty intervals provided by the Cases I-VIII that guarantee that when f'_{x_0} belongs to them, then the fixed point $\mathbf{x}_0$ is stable:

$$f'_{x_0} \in \left(-1, -\frac{e^{-N\xi}}{1-e^{-\xi}}\right) \cup \left(\max\left\{-1, -\frac{e^{-N\xi}}{1-e^{-\xi}}, -\frac{1-e^{-N\xi}}{|1-2e^{-\xi}+e^{-N\xi}|}\right\}, -\frac{e^{-\xi}-e^{-N\xi}}{|2-3e^{-\xi}+e^{-N\xi}|}\right) \cup \left(\max\left\{-1, -\frac{e^{-N\xi}}{1-e^{-\xi}}, -\frac{e^{-\xi}-e^{-N\xi}}{|2-3e^{-\xi}+e^{-N\xi}|}\right\}, 0\right) \cup [0,1). \quad \text{(D.64)}$$

Now, it is easy to see that

$$\max\left\{-1, -\frac{e^{-N\xi}}{1-e^{-\xi}}, -\frac{e^{-\xi}-e^{-N\xi}}{|2-3e^{-\xi}+e^{-N\xi}|}\right\} \geq -1,$$
$$\max\left\{-1, -\frac{e^{-N\xi}}{1-e^{-\xi}}, -\frac{1-e^{-N\xi}}{|1-2e^{-\xi}+e^{-N\xi}|}\right\} \geq -1,$$

and hence the condition (D.64) reduces to

$$|f'_{x_0}| < 1.$$

Notice that the proof remains valid for the case of (not necessary asymptotic) stability in the statement of the theorem. In this case we require

$$\rho(A(\mathbf{x}_0, \boldsymbol{\theta})) \leq |||A(\mathbf{x}_0, \boldsymbol{\theta})|||_\infty = \max_{1\leq i\leq N} \sum_{j=1}^{N} |a_{ij}| \leq 1,$$

which results in the condition

$$|f'_{x_0}| \leq 1,$$

as required. □

The following theorem provides the characteristic polynomial of the connectivity matrix $A(\mathbf{x}_0, \boldsymbol{\theta})$ and an explicit expression for its spectral radius under some conditions. Another way to find upper bounds for this spectral radius would consist of using the Cauchy bound [10] of this polynomial. In our experience this approach produces mediocre results in comparison with the statement in Theorem D.10.

Theorem D.11 *Let $A(\mathbf{x}_0, \boldsymbol{\theta})$ be the connectivity matrix of the reservoir map in (B.4). Define $\Phi := (1-e^{-\xi})f'_{x_0}$ and let $\{\lambda_1, \ldots, \lambda_N\}$ be the roots of the polynomial equation*

$$\lambda^N - \left(\frac{e^{-N\xi}}{\Phi} + N\right)\lambda^{N-1} + \sum_{j=0}^{N-2} \binom{N}{j} (-1)^{N-j}\lambda^j = 0. \quad \text{(D.65)}$$

Then $\rho(A(\mathbf{x}_0, \boldsymbol{\theta})) = \max\{|\Phi\lambda_1|, \ldots, |\Phi\lambda_N|\}$. Moreover, if (D.65) has a root λ such that $\lambda > 1$, then $\rho(A(\mathbf{x}_0, \boldsymbol{\theta})) = \lambda|\Phi|$ necessarily.

Proof. Define first $B := \frac{1}{\Phi}A(\mathbf{x}_0, \boldsymbol{\theta})$. We then write

$$B := \begin{pmatrix} 1 & 0 & \ldots & 0 & \frac{e^{-\xi}}{\Phi} \\ e^{-\xi} & 1 & \ldots & 0 & \frac{e^{-2\xi}}{\Phi} \\ e^{-2\xi} & e^{-\xi} & \ldots & 0 & \frac{e^{-3\xi}}{\Phi} \\ \vdots & \vdots & \vdots & & \vdots \\ e^{-(N-1)\xi} & e^{-(N-2)\xi} & \ldots & e^{-\xi} & 1+\frac{e^{-N\xi}}{\Phi} \end{pmatrix}.$$

Let $\mathbf{v} = (v_1, \ldots, v_N)$ be an eigenvector of B with eigenvalue λ, that is, $B\mathbf{v} = \lambda\mathbf{v}$. This equality can be rewritten as

$$\begin{cases} v_1 & = & \frac{e^{-\xi}}{(\lambda-1)\Phi} v_N, \\ v_2 & = & \frac{e^{-2\xi}}{(\lambda-1)\Phi} v_N \frac{\lambda}{\lambda-1}, \\ v_3 & = & \frac{e^{-3\xi}}{(\lambda-1)\Phi} v_N \frac{\lambda^2}{(\lambda-1)^2}, \\ \vdots & \vdots & \vdots \\ v_{N-1} & = & \frac{e^{-(N-1)\xi}}{(\lambda-1)\Phi} v_N \frac{\lambda^{N-2}}{(\lambda-1)^{N-2}}, \end{cases} \tag{D.66}$$

together with the identity

$$\sum_{j=1}^{N} b_{Nj} v_j = \lambda v_N,$$

which is equivalent to

$$\frac{v_N}{(\lambda-1)\Phi} e^{-N\xi} \left(1 + \frac{\lambda}{\lambda-1} + \frac{\lambda^2}{(\lambda-1)^2} + \cdots + \frac{\lambda^{N-2}}{(\lambda-1)^{N-2}}\right) + v_N \left(1 + \frac{e^{-N\xi}}{\Phi}\right) = \lambda v_N,$$

or, equivalently, to

$$v_N \left(\frac{e^{-N\xi}}{\Phi} \frac{\lambda^{N-1} - (\lambda-1)^{N-1}}{(\lambda-1)^{N-1}} + (\lambda-1) + \frac{e^{-N\xi}}{\Phi} \right) = 0.$$

If we assume that $b_N \neq 0$ and $\lambda \neq 1$, this amounts to

$$\frac{(\lambda-1)^N}{\lambda^{N-1}} = \frac{e^{-N\xi}}{\Phi}.$$

Consequently, the eigenvalues of A are given by the roots $\{\lambda_1, \ldots, \lambda_N\}$ of the polynomial equation

$$\lambda^N - \left(\frac{e^{-N\xi}}{\Phi} + N\right) \lambda^{N-1} + \sum_{j=0}^{N-2} \binom{N}{j} (-1)^{N-j} \lambda^j = 0$$

and hence

$$\rho(A(\mathbf{x}_0, \boldsymbol{\theta})) = \max\{|\Phi\lambda_1|, \ldots, |\Phi\lambda_N|\}.$$

If there exists some $\lambda > 1$, the expressions (D.66) show that the eigenvector $\mathbf{v}$ of B (or of $-B$ if $\Phi < 0$) can be chosen positive. In that situation Corollary 8.1.30 in [9] guarantees that $\rho(B) = \lambda$ with $\lambda > 1$ the eigenvalue corresponding to $\mathbf{v}$ and hence $\rho(A(\mathbf{x}_0, \boldsymbol{\theta})) = |\Phi\lambda|$ as required. □

E Robustness of the empirical tests with respect to the choice of nonlinear kernel

In this section we show the robustness of the empirical results in Sections 1.1 and 2 of the paper with respect to the choice of the nonlinear kernel used in the construction of the TDR.

First, in Section 1.1 we carried out an experiment using the Ikeda kernel and a quadratic memory task that showed that optimal performance is obtained when the input mean and variance are tuned so that the dynamics of the reservoir takes place in the neighborhood of a stable steady state and

making sure that multimodality is avoided. We have repeated here the same experiment but, this time, using the Mackey-Glass kernel. More specifically, we nonsider a TDR with $N = 20$ neurons $d = 0.943$, $\gamma = 4.7901$, $\eta = 1.3541$, and $p = 2$. As we explained in Corollary D.6, with these parameter values $x_0 = -\sqrt{\eta - 1} = -0.5951$ is an equilibrium that satisfies the sufficient conditions for asymptotic stability. In order to verify that the optimal performance is obtained when the RC operates in a neighborhood of that stable equilibrium, we study the normalized mean square error (NMSE) exhibited by a TDR initialized at $x_0 = -0.5951$ when we present to it a quadratic memory task. More specifically, we inject in a TDR under study an independent and identically normally distributed signal $z(t)$ with mean zero and variance 10^{-4} and we then train a linear readout $W_{\rm out}$ (obtained with a ridge penalization of $\lambda = 10^{-15}$) in order to recover the quadratic function $z(t-1)^2 + z(t-2)^2 + z(t-3)^2$ out of the reservoir output. The top left panel in Figure 2 shows how the NMSE behaves as a function of the mean and the variance of the input mask $\mathbf{c}$. It is clear that by modifying any of these two parameters we control how far the reservoir dynamics separates from the stable equilibrium, which we quantitatively evaluate in the two bottom panels by representing the RC performance in terms of the mean and the variance of the resulting reservoir output. Both panels depict how the injection of a signal slightly shifted in mean or with a sufficiently high variance results in reservoir outputs that separate from the stable equilibrium and in a severely degraded performance. An important factor in this deterioration seems to be the multi modality, that is, if the shifting in mean or the input signal variance are large enough then the reservoir output visits the stability basin of the other stable point placed at $x_0 = \sqrt{\eta - 1} = 0.5951$; in the top right and bottom panels we have marked with red color the values for which bimodality has occurred so that the negative effect of this phenomenon is noticeable.

Second, in Section 2 we used a Mackey-Glass based reservoir to compare the empirical performance surfaces in terms of various parameters with that coming from the formula (C.16) that was obtained as a result of modeling the reservoir with an approximating VAR(1) process. In this section we have repeated the same exercise with an Ikeda based reservoir in order to show that the formula (C.16) produces in this case results of comparable quality. The outcome of this experiment are contained in Figure 3 where we represent the normalized mean square error as a function of the distance between neurons and the feedback gain η. The other fixed parameter values used are $\gamma = 0.523$ and $\phi = 0.3106$; the reservoir was constructed using 20 neurons and we presented to it the three-lag quadratic memory task corresponding to the diagonal matrix Q with diagonal entries given by the vector $(0, 1, 1, 1)$. The optimal output mask $\mathbf{W}_{\rm out}$ was computed using a ridge regression with $\lambda = 10^{-15}$. As it can be seen in the figure, we restrict the values of the parameter η to the interval $[0, 1]$ which ensures, using one of the results in Corollary D.7, that the TDR exhibits for each value of η a unique equilibrium (unimodality is hence guaranteed) that is always stable. The TDR is always initialized at that stable configuration.

References

[1] Grigoryeva, L., Henriques, J., Larger, L. & Ortega, J.-P. Optimal nonlinear information processing capacity in delay-based reservoir computers. *Preprint* (2014).

[2] Lütkepohl, H. *New Introduction to Multiple Time Series Analysis* (Springer-Verlag, Berlin, 2005).

[3] Tikhonov, A. N. On the stability of inverse problems. *Dokl. Akad. Nauk SSSR* **39**, 195–198 (1943).

[4] Hale, J. *Theory of Functional Differential Equations* (Springer-Verlag, 1977).

[5] Wu, M., He, Y. & She, J.-H. *Stability Analysis and Robust Control of Time-Delay Systems* (Springer, 2010).

[6] Krasovskiy, N. N. *Stability of Motion* (Stanford University Press, 1963).

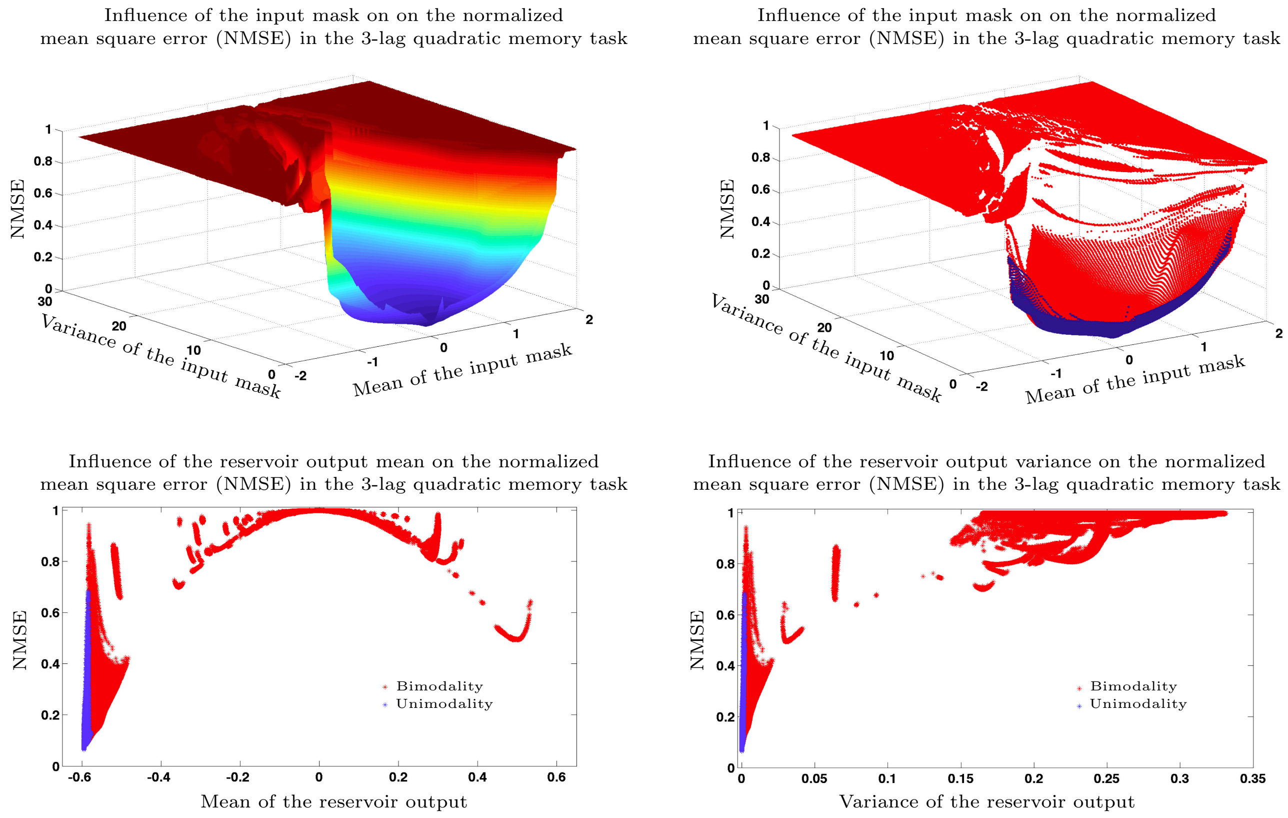

Figure 2: Behavior of the performance of a Mackey-Glass based reservoir in a quadratic memory task as a function of the mean and the variance of the input mask. The modification of any of these two parameters influences how the reservoir dynamics separates from the stable equilibrium. The top panels show how the performance degrades very quickly as soon as the mean and the variance of the input mask (and hence of the input signal) separate from zero. The bottom panels depict the reservoir performance as a function of the various output means and variances obtained when changing the input means and variances. In the top right and bottom panels we have indicated with red markers the cases in which the reservoir visits the stability basin of a contiguous equilibrium hence showing how unimodality is associated to optimal performance.

[7] Mackey, M. C. & Glass, L. Oscillation and chaos in physiological control systems. *Science* **197**, 287–289 (1977).

[8] Ikeda, K. Multiple-valued stationary state and its instability of the transmitted light by a ring cavity system. *Optics Communications* **30**, 257–261 (1979).

[9] Horn, R. A. & Johnson, C. R. *Matrix Analysis* (Cambridge University Press, 2013), second edn.

[10] Rahman, Q. I. & Schmeisser, G. *Analytic Theory of Polynomials* (Clarendon Press, Oxford, 2002).

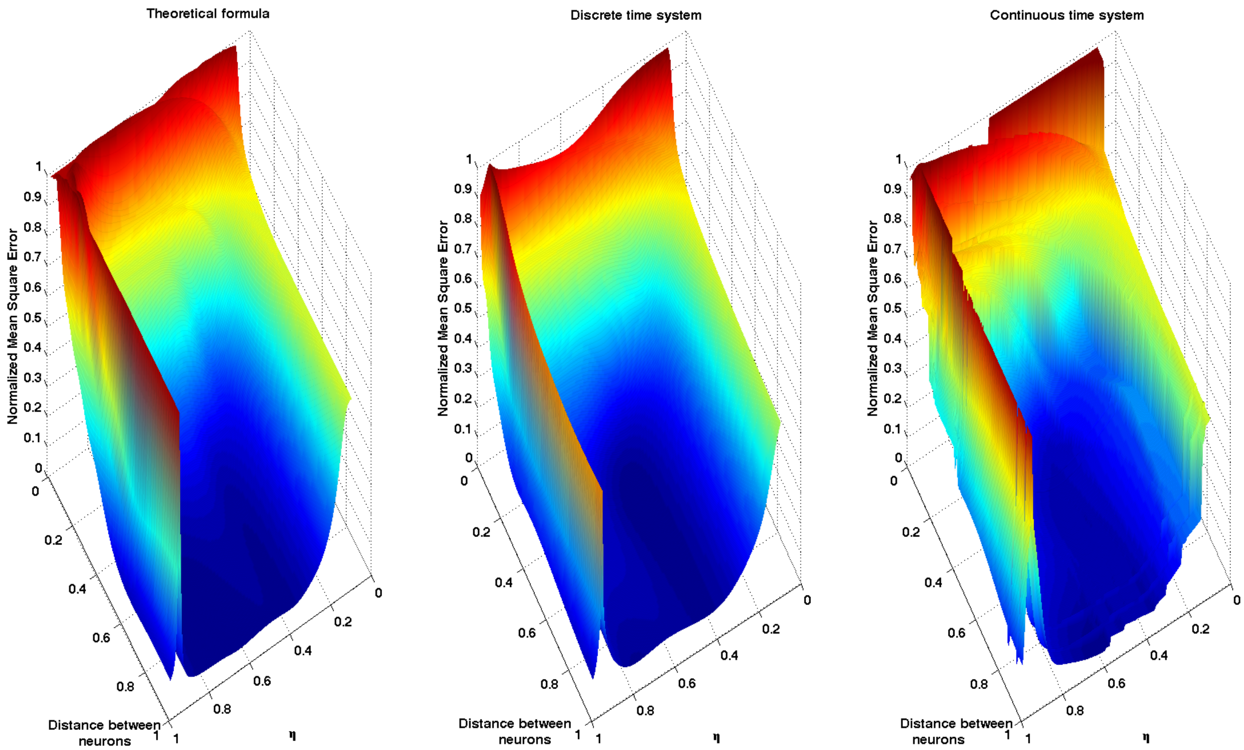

Figure 3: Error surfaces exhibited by an Ikeda based reservoir computer in a 3-lag quadratic memory task, as a function of the distance between neurons and the parameter η. The points in the surfaces of the middle and right panels are the result of Monte Carlo evaluations of the NMSE exhibited by the discrete and continuous time TDRs, respectively. The left panel was constructed using the formula (C.16) that is obtained as a result of modeling the reservoir with an approximating VAR(1) process.
